# Phylloquinone improves endothelial function, inhibits cellular senescence, and vascular inflammation

**DOI:** 10.1007/s11357-024-01225-w

**Published:** 2024-07-09

**Authors:** Anna Kieronska-Rudek, Agnieszka Kij, Anna Bar, Anna Kurpinska, Tasnim Mohaissen, Marek Grosicki, Marta Stojak, Magdalena Sternak, Elżbieta Buczek, Bartosz Proniewski, Kamil Kuś, Joanna Suraj-Prazmowska, Agnieszka Panek, Monika Pietrowska, Szczepan Zapotoczny, Catherine M. Shanahan, Csaba Szabo, Stefan Chlopicki

**Affiliations:** 1 Jagiellonian Centre for Experimental Therapeutics (JCET), Jagiellonian University, Krakow, Poland; 2 Chair of Pharmacology, Faculty of Medicine, Jagiellonian University Medical College, Krakow, Poland; 3 Chair of Pharmacology, Faculty of Science and Medicine, University of Fribourg, Fribourg, Switzerland; 4 Institute of Nuclear Physics Polish Academy of Sciences, Krakow, Poland; 5 Centre for Translational Research and Molecular Biology of Cancer, Maria Sklodowska-Curie National Research Institute of Oncology, Gliwice, Poland; 6 Department of Physical Chemistry and Electrochemistry, Faculty of Chemistry, Jagiellonian University, Krakow, Poland; 7 School of Cardiovascular and Metabolic Medicine and Sciences, James Black Centre, King’s College London, London, UK

**Keywords:** Vitamin K_1_, Vitamin K_2_, Vasoprotection, Anti-senescence, Anti-inflammatory, DNA damage

## Abstract

Phylloquinon (PK) and menaquinones (MK) are both naturally occurring compounds belonging to vitamin K group. Present study aimed to comprehensively analyze the influence of PK in several models of vascular dysfunction to determine whether PK has vasoprotective properties, similar to those previously described for MK. Effects of PK and MK on endothelial dysfunction were studied in ApoE/LDLR^−/−^ mice in vivo, in the isolated aorta incubated with TNF, and in vascular cells as regard inflammation and cell senescence (including replicative and stress-induced models of senescence). Moreover, the vascular conversion of exogenous vitamins to endogenous MK-4 was analyzed. PK, as well as MK, given for 8 weeks in diet (10 mg/kg) resulted in comparable improvement in endothelial function in the ApoE/LDLR^−/−^ mice. Similarly, PK and MK prevented TNF-induced impairment of endothelium-dependent vasorelaxation in the isolated aorta. In in vitro studies in endothelial and vascular smooth muscle cells, we identified that both PK and MK displayed anti-senescence effects via decreasing DNA damage while in endothelial cells anti-inflammatory activity was ascribed to the modulation of NFκB activation. The activity of PK and MK was comparable in terms of their effect on senescence and inflammation. Presence of endogenous synthesis of MK-4 from PK in aorta and endothelial and smooth muscle cells suggests a possible involvement of MK in vascular effects of PK. In conclusion, PK and MK display comparable vasoprotective effects, which may be ascribed, at least in part, to the inhibition of cell senescence and inflammation. The vasoprotective effect of PK in the vessel wall can be related to the direct effects of PK, as well as to the action of MK formed from PK in the vascular wall.

**Supplementary Information:**

The online version contains supplementary material available at 10.1007/s11357-024-01225-w.

## Introduction

Vitamin K is a group of homologs that include the naphthoquinone ring in their structure. The analogs, which consist of side isoprenoid chains, have been divided into two groups: saturated PK, phylloquinone, and unsaturated vitamers of MK named menaquinones (MK-n), where “n” means the number of isoprenyl units in the side chain of MK [1]. PK is primarily synthesized by plants and represents a major dietary source of vitamin K, while MK is produced endogenously by the intestinal microbiota and is contained mainly in dietary animal-derived products or in fermented foods such as “natto.”

The main function of vitamin K is to act as a cofactor for glutamyl carboxylase (GGCX)—an enzyme participating in the carboxylation of multiple, vitamin K–dependent proteins (VKDP). Although PK and MK share many properties, PK has been mostly recognized as a key factor involved in the carboxylation of coagulation factors and as a regulator of coagulation, primarily acting in the liver [2]. In contrast, MK has been proposed as an extrahepatically active compound, participating in the regulation of calcium metabolism by carboxylation of VKDP such as Matrix Gla Protein (MGP), Growth Arrest Specific 6 (GAS6), and osteocalcin [3–5].

The division of the roles of PK and MK described in the literature for hepatic and extrahepatic functions, respectively, has been challenged by various emerging sets of data. Firstly, MK and PK have been shown to have a comparable affinity to vitamin K epoxide reductase (VKOR), an enzyme participating in vitamin K–dependent carboxylation [6]; thus, both MK and PK may contribute to post-translation carboxylation of vitamin K–dependent proteins (VKDP). Secondly, several studies conducted in the last decade provide evidence that apart from canonical activity related to participation in VKD carboxylation, vitamin K displays a number of non-canonical activities, for example, regulating the activity of transcriptional factors SXR and NF-κB [7, 8] or acting as an electron carrier [9]. Some of these activities are shared by PK and MK. Finally, although previously PK activity was limited to the liver, several extrahepatic functions of PK have been described, including effects on vascular function, [10, 11] blood cholesterol level [12], and inflammation [8, 13, 14].

Interestingly, evolutionary evidence shows that partially saturated menaquinones have been identified in bacteria [15, 16]. These findings suggest that PK synthesis by plant chloroplasts most likely evolved by endosymbiosis with cyanobacteria [17] indicating that plant PK could be possibly interconverted to MK-4. In fact, many types of human cells and tissues such as gut microbiota, kidneys, intestines, brain, macrophages, and endothelial cells [14, 18–23] were reported to produce endogenous MK-4 from dietary/exogenous PK. The most commonly accepted model of endogenous synthesis of MK-4 assumes removing the side chain of vitamin dietary PK and then prenylation of menadione via statin-sensitive UBIAD-1, the enzyme involved in intracellular de novo cholesterol synthesis [14, 22–24]. Recent data also demonstrate that endogenous MK-4 can be generated not only from PK but also from MK-5 in macrophages [14]. Thus, interconversion of various forms of vitamin K may be more prevalent than previously thought and there may be more flexibility at the cellular level to maintain vitamin K–dependent function depending on the availability of various exogenous substrates.

Considering the possibility of extra-hepatic activity of PK and the recent data showing improvement of endothelial function by MK-7 an effect that was not related to previously described effects of MK-7 on calcification [25], we hypothesized that PK may have vasoprotective effects and the current experiments were set up to test this hypothesis. Therefore, we comprehensively analyze the influence of PK in several models of vascular dysfunction to determine whether PK has vasoprotective properties, similar to those previously described for MK.

In the present work, we demonstrated that PK exerts vasoprotective effects comparably to MK. These results contribute to the formulation of a new paradigm, whereby dietary PK emerges as an important nutritional protective factor against the development of vascular dysfunction and aging of the cardiovascular system, the activities no longer reserved for MK only [26, 27].

## Materials and methods

### Animals, cell lines, cell culture, and reagents

In vivo studies were performed on 16–25-week-old mice. The ApoE/LDLR^−/−^ female mice were provided by the Department of Human Nutrition, the University of Agriculture in Krakow (Krakow, Poland); C57BL/6 male mice (Mossakowski Medical Research Centre, Polish Academy of Sciences, Warsaw, Poland and Medical University, Warsaw, Poland). Throughout the experiment, animals were housed 5–6 mice per cage, with a 12 h light/day cycle, and given unlimited access to food.

The ApoE/LDLR^−/−^ mice, a model of endothelial dysfunction and atherosclerosis, [28–30] were fed for 8 weeks with a standard diet described in detail [25] or a diet supplemented with PK or MK at a dose of 10 mg/kg b.w./day.

After 8 weeks of PK/MK-enriched diet, the endothelial-related response to acetylcholine (Ach) was measured in the brachiocephalic artery (BCA) and left coronary artery (LCA) using the MRI method as described in detail below. Mice were sacrificed using a mixture of ketamine and xylazine in doses of 100 and 10 mg/kg b.w./day, respectively. The experiments involving animals were conducted after obtaining permission from the II Local Ethical Committee on Animal Testing in the Institute of Pharmacology, Polish Academy of Sciences, and performed according to the guidelines from Directive 2010/63/EU of the European Parliament on the protection of animals used for scientific purposes.

For in vitro studies, human aortic endothelial cells (HAEC), murine aortic smooth muscle cells (MOVAS), and U937 human monocytic cells were purchased at ATTC (Rockville, Maryland, MD, USA). Porcine aortic endothelial cells (PAEC) were a gift from Professor Yu Wang (University of Hong Kong). Primary human aortic smooth muscle cells from a 35-year-old female (04/35F/11A) were collected from the ascending aorta of a transplant donor with appropriate ethical permission. Cells were cultured in supplemented medium: MOVAS—Dulbecco’s Modified Eagle Medium, DMEM (Gibco, Scotland, UK), containing 10% FBS, U937—RPMI 1640 (Lonza, Basel, Switzerland) containing 10% FBS, PAEC—DMEM containing 20% FBS, and 04/35F/11A—Medium 199 (Sigma-Aldrich, UK) containing 20% FBS.

HAEC—growth media EBM-2 (Lonza, Basel, Switzerland) with supplement kit recommended by the manufacturer (Lonza, Basel, Switzerland). Cells were cultured at 37 °C, 5% CO_2_, and regularly tested for mycoplasma contamination using the MycoAlert Mycoplasma Detection Kit (Lonza, Basel, Switzerland).

PK, MK-4, and MD were purchased from Sigma-Aldrich (MO, USA). MK-7 was provided by NattoPharma, (NattoPharma, part of Gnosis by Lesaffre, Lesaffre, France). Deuterium-labeled vitamins MD, PK, and MK-7 were purchased from Sigma-Aldrich (MO, USA). All experiments were conducted in the dark. In experiments longer than 24 h, the vitamin K–supplemented medium was changed once daily to ensure the presence of fresh vitamin K.

### Collection of biological material plasma, aorta, and liver

Blood samples were collected using EDTA-coated tubes and centrifuged to separate plasma, which was collected for further analysis. The aortas were isolated and placed in cold NaCl or Krebs–Henseleit solution (KB) bubbled with a 95% O_2_/5% CO_2_ mixture (pH = 7.4) to remove surrounding tissues, including perivascular adipose tissue.

Livers were perfused using NaCl, and subsequently, two liver lobes were collected and frozen for vitamin K content analysis, while the rest of the liver was collected in formalin for histology.

### Studies of endothelial function in vivo, using Magnetic Resonance Imaging (MRI)

The effect of PK or MK (MK-4) on endothelial function was assessed using magnetic resonance imaging (MRI) using a 9.4 T scanner (BioSpec 94/20 USR; Bruker, Germany) according to protocols described previously [31–34]. During the experiment, mice were under isoflurane anesthesia (1.5% in the mixture of oxygen and air 1:2). The body temperature, heart activity, and respiration were constantly monitored by the Monitoring and Gating System (SA Inc, Stony Brook, NY, USA).

Endothelial function in vivo was assessed based on endothelium-dependent response to acetylcholine (Ach) (Sigma-Aldrich, Poznan, Poland 16.6 mg/kg b.w., *i.p*), while vascular smooth muscle cell–dependent response was measured in response to sodium nitroprusside (SNP) (Sigma-Aldrich, Poznan, Poland 1 mg/kg b.w., *i.v*) in BCA and LCA. The doses of Ach and SNP as well as the route of their administration were chosen based on our previous study whereby protocol of endothelial function assessment in vivo by MRI was established [35] Of note, at the time of MRI measurement (25–30 min after Ach administration), Ach did not induce alterations in hemodynamics and in cardiac rhythm.

Images were acquired using the cine IntraGateTM FLASH 3D sequence and reconstructed with the IntraGate 1.2.b.2 macro-Bruker (Bruker, Germany). The quantitative analysis was performed in ImageJ software 1.46r (NIH, Bethesda, MD, USA) and scripts written in Matlab (MathWorks, Natick, MA, USA) as previously described [31, 32].

### Studies of endothelial function and nitric oxide production in isolated aorta

The isolated aorta was cut longitudinally (for NO measurements) or cut into rings (for myography) and incubated for 24 h with shaking in MEM supplemented with 20% FBS, containing 10 ng/mL TNF in the presence or absence of 10 μM PK or MK. For comparison, aortas were incubated in a non-supplemented medium without TNF.

To study the functional responses in the aorta, the aortas were cleaned in cold Krebs–Henseleit (KB) solution purged with 95% O_2_ and 5% CO_2_ (pH = 7.4), and then cut into rings. The influence of PK and MK (MK-7) on isolated, TNF-stimulated [10 ng/mL] aorta was analyzed using the Mulvany myograph system (620 M, Danish Myo Technology, Denmark). The endothelium-dependent and endothelium-independent response in isolated aorta was assessed according to the previously described protocols [36, 37]. Briefly, the aortic rings were initially preconstricted with phenylephrine, and then the vasorelaxant response to Ach (0.01–10 μM), reflecting an endothelium-dependent response, and the vasorelaxant response to SNP (0.001–1 μM), as an endothelium-independent response, were measured.

To study the effects of vitamin K treatment on NO production, the EPR method was used as described previously [36, 38]. Measurements were performed in the presence of L-NIL (Cayman, MI, USA) to inhibit iNOS-dependent NO generation. NO was measured using EPR spin trapping with diethyldithiocarbamate acid sodium salt (DETC, Sigma-Aldrich, Poznan, Poland). Before the measurement, the aorta was weighed, and NO production was normalized to the aortic weight.

## Analysis of the anti-senescence activity of PK and MK

### Replicative cell senescence in PAEC and 04/35F/11A cells

Porcine aortic endothelial cells (PAEC) and human aortic smooth muscle cells 04/35F/11A were used to assess the effect of PK and MK on the process of cell replication. Cell senescence in the replicative senescence model was achieved by sequential passages resulting in multiple cell replication. The phenotype of PAEC cells was considered senescent after the fifth passage, and in the case of 04/35F/11A after the twenty-first passage. Cells were then treated for 24 h (PAEC) or 48 h (04/35F/11A) with PK or MK (MK-7) at a concentration of 5 or 10 µM. Subsequently, the proliferative potential and senescence-associated β-galactosidase activity (SA-β-gal) was assessed as described below.

### X-ray-induced cell senescence

The MCN 323 apparatus (Philips, Hamburg, Germany) with parameters of 250 kV and 10 mA was used for HAEC irradiation. Dosimetry was performed using the reference UNIDOS dosimeter and the TM31013 ionization chamber (PTW, Freiburg, Germany). The dimensions of the radiation field were estimated at 20 × 20 cm^2^, and the distance between the source and the surface was 34.8 cm [39]. Culture bottles with the HAEC line were irradiated on a specially designed polymethyl methacrylate phantom. The average measured dose rate was 0.03 Gy/s and the total dose delivered to the samples was 10 Gy. After exposure, cells were transferred to a cell incubator. Subsequently, 24 h after irradiation, the culture medium was replaced with a fresh medium containing PK or MK (10 µM), or a control medium without vitamins. The cell senescence process after irradiation was estimated to be 10 days, by the analysis of proliferation and SA-β-gal activity. In the experimental period of 10 days, the medium was changed daily. The influence of PK and MK on cell senescence and radiation-induced DNA damage was assessed by proteomic analysis.

### Prelamin A accumulation-induced cell senescence

Senescence of 04/35F/11A vascular smooth muscle cells was achieved in two ways: by overexpressing prelamin A with an adenoviral vector, or by silencing the Farnesylated Protein Converting Enzyme-1 (FACE-1) gene with siRNA, resulting in the inhibition of prelamin A maturation and, consequently, its accumulation.

The adenovirus transfection procedure was started when the confluence of 04/35F/11A cells was 60–70%. The viral titer used for transfection was approximately 40 virus units per cell. Cells were infected with FLAG-tagged recombinant adenoviruses containing a non-cleavable form of prelamin A modified at the cleavage site of Zmpste24 (L647R) (Ad/UC) [40]. In the present study, the pShuttle2 (cloning vector) designed for use with the Adeno-X™ Expression System was used (Takara bio, USA, CA; cat. 631,513 & 631,022). Transfection efficiency was assessed by immunocytochemical staining with an anti-FLAG antibody (Sigma-Aldrich, Saint Louis, MO, USA). The expected efficiency of transfection was about 80%.

Incubation with PK and MK (10 µM) was started 40 min after the virus introduction and was carried out for 48 h. The medium was changed every 24 h. The senolytic effect of PK and MK was assessed based on the analysis of gene expression related to cell aging and SA-β-gal activity.

The FACE-1 expression silencing procedure was started at 70–80% 04/35F/11A confluence. FACE-1 small interference RNA (siRNA) at 5 nM was introduced into the cells using HiPerfect transfection reagent (Qiagen, Manchester, UK). The efficiency of transfection has been estimated by the level of γH2A.X expression FACE-1 transfected cells compared to the scrambled control (Supplementary Fig. 1). Vitamin K treatment was initiated 24 h after transfection by changing the culture medium to a medium supplemented with 10 µM PK or MK. Incubation was carried out for 24 h.

FACE 1 is a zinc metallopeptidase responsible for the enzymatic cleavage of terminal residues of farnesylated prelamin A to form mature lamin A. The mechanisms whereby the accumulation of prelamin A can induce DNA damage are multiple and include global downregulation of transcription of DNA repair factors [41] interference with nuclear import or DNA repair factors such as 53BP1 [42] and induction of mono-ubiquitination of PCNA followed by induction of Pol η, resulting in DNA replication fork stalling, collapse of DNA replication fork, and initiation of DNA double-strand breaks [43, 44]. Double-strand breaks can be detected by the expression of phosphorylated yH2A.x, an early marker of the cellular response to DNA double-strand breaks. Therefore, to study the influence of PK and MK on prelamin A accumulation-induced DNA damage in the model of FACE-1 knockout, the expression of phosphorylated histone γH2A.X (Ser139) (Cell Signaling Technology, UK) was measured.

### Analysis of SA-β-gal activity

To confirm the phenotype of senescent cells as well as the anti-senescence effect of PK and MK (MK-7), SA-β-gal activity was determined using commercially available tests (Cell Signaling Technology, Great Britain or Sigma Aldrich, Poland) according to the manufacturer’s protocol. Staining was carried out overnight in a cell culture incubator in a humid atmosphere at 37 °C, without CO_2_.

### Analysis of changes in gene expression associated with cell senescence by RT-PCR

Changes in gene expression associated with senescence were determined using quantitative real-time polymerase chain reaction (RT-PCR) with reverse transcription as described in detail in Supplementary Methods.

### Proteomic analysis of changes in protein profile associated with cell senescence

Label-free non-targeted differential proteomic analysis was performed to assess protein profile changes associated with HAEC senescence in the X-ray-induced aging model.

Cells were washed twice with PBS and lysed in lysis buffer containing 7 M urea (BioShop, Burlington, Ontario, Canada), 2 M thiourea (BioShop, Burlington, Ontario, Canada), and 30 mM Tris–HCl (pH 8.0) (BioShop, Burlington, Ontario, Canada), sonicated on ice, and centrifuged (16,000 × *g*, 15 min, 4 °C), and the supernatants were collected. Protein concentration was assessed using Bradford protein assay (Bio-Rad, Hercules, CA, USA) following the manufacturer’s protocol. Cells were prepared according to the modified FASP protocol [45]. For the detailed protocol, see Supplementary Methods.

### Analysis of the effects of PK and MK on proliferation of senescent cells

Changes in cell proliferation were assessed in HAEC by the wound healing assay using the electric cell-substrate impedance sensing (ECIS) method or by manual cell counting using a Bürker chamber. A detailed description of the ECIS method can be found in the Supplementary Methods.

## Analysis of the anti-inflammatory activity of PK and MK

### Measurement of prostaglandin E_2_ (PGE_2_) production

The influence of PK and MK (MK-7; 10 μM) on PGE_2_ production in TNF-stimulated HAEC cells was measured using commercially available enzyme-linked immunosorbent assay (ELISA) enzyme-linked immunosorbent assay PGE_2_—Elisa Kit (ENZO, Lausanne, Switzerland) according to manufacturer protocol.

### Immunocytochemical analysis

Analysis of ICAM, COX-2, NF-κB, and Phospho-Histone γH2A.x protein expression based on immunocytochemical staining with the automatic image was done using Columbus v. 2.4.2 software (PerkinElmer, Waltham, MA, USA) as described in detail in Supplementary Methods.

### Cell adhesion assay

Adhesion assay was performed at static conditions. Confluent HAEC were treated with PK or MK (MK-7) at 0.1–10 µM concentration for 48 h. Simultaneously, U937 cells were treated with PK or MK in the same arrangements as endothelial cells 24 h before the actual test, and HAEC was activated with 10 ng/mL TNF (Sigma-Aldrich, Saint Louis, MO, USA). Before the cellular adhesion, U937 cells were washed 1 × with DPBS and loaded with 1 µM Calcein AM solution (Sigma-Aldrich, Saint Louis, MO, USA) for 30 min. Calcein-stained U937 cells, as well as stimulated HAEC, were washed 1 × with DPBS. Afterward, U937 cells were added to microplate wells at a density of 2 × 10^4^ cells per well and were allowed to adhere to an endothelial monolayer for 30 min at 37 °C, 5% CO_2_. After incubation, cells were washed gently 2 × with DPBS to eliminate non-adherent cells. The remaining cells were stained against their nuclei with Hoechst 33,342. Microscopic images from 6 microscopic fields were acquired using a Yokogawa CQ1 High-Content Analysis System (excitation lasers 405/488 nm and emission filters 452/45 and 525/50). Images were analyzed in Columbus 2.4.2 (PerkinElmer, Waltham, MA, USA) software. Adherent U937 cells were identified based on the high fluorescence of the cell nucleus and FITC channel.

## Analysis of endogenous MK-4 content in endothelial cells, vascular smooth muscle cells, and the isolated aorta incubated with PK or MK

To study the content of MK-4 in cells (HAEC or MOVAS) or isolated aorta samples in the presence or absence of PK, MK (MK-7), or menadione (MD) for comparison, cells or aorta were incubated for 24 h. To verify whether MK-4 synthesis from MD was mediated by UBIAD-1 [22, 23] prenylation was inhibited by atorvastatin (ATO) 1 μM, added together with MD for 24 h incubation.

The concentration of PK that was taken up into the cells and the concentration of the endogenously synthesized MK-4 was measured using the UHPLC-MS/MS method as described previously [14, 25]. Calibration curves were plotted as the relationship between the peak area ratios of the analyte/internal standard to the nominal concentration of the analyte. The levels of various forms of vitamin K were calculated based on the regression equations generated for each analyte. The quantification of MK-4-d_7_ production from exogenous PK-d_7_, MK-7-d_7_, and K_3_-d_8_ was performed based on the regression equations calculated for MK-4.

## Statistical analysis

Statistical analysis was performed using GraphPad Prism 10 (San Diego, CA, USA). Depending on data distribution (tested with the Shapiro–Wilk or D’Agostino–Pearson omnibus normality test), data were presented as mean ± SD, mean ± SEM, or median ± IQR, respectively, of at least three biological experiments, with a minimum of three technical replicates. The statistical significance was defined in the post hoc tests; *p*-values < 0.05 were considered statistically significant.

## Results

### PK and MK reversed endothelial dysfunction in ApoE/LDLR^−/−^ mice in vivo

**Fig. 1:**
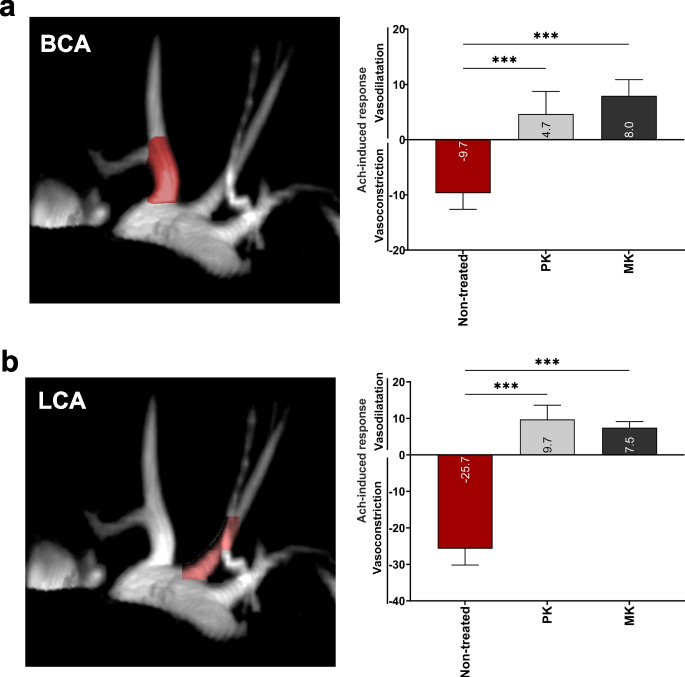
PK and MK improve endothelial function in vivo. The effect of an 8-week PK or MK (MK-4)-supplemented diet (10 mg/kg) on endothelium-dependent vasodilatation was assessed in a brachiocephalic artery (BCA) (**a**) and left coronary artery (LCA) (**b**) in ApoE LDLR^−/−^ mice in in vivo using MRI. Results are presented as mean ± SD *n* = 6–10. Statistical analysis was performed using one-way or two-way ANOVA with Dunnett’s multiple comparisons. The symbol *** indicates statistical significance at *p* < 0.001, respectively. The effects of MK (MK-7) were previously published.^23^

ApoE/LDLR^−/−^ mice represent a commonly used model of hypercholesterolemia, where animals spontaneously develop endothelial dysfunction [46] followed by atherosclerotic plaques over time [47]. Earlier studies conducted in our laboratory demonstrated that 8 weeks of feeding with the diet supplemented with MK (MK-7, 10 mg/kg) resulted in an improvement of endothelial function, measured with MRI in 24-week-old ApoE/LDLR^−/−^ mice, when compared to ApoE/LDLR^−/−^ mice fed with a standard control diet [25]. In the present study, using the identical experimental approach, we demonstrated that in ApoE/LDLR^−/−^ mice fed with a diet supplemented with PK (10 mg/kg) or with MK (MK-4; 10 mg/kg), endothelial function was improved. In non-treated ApoE/LDLR^−/−^ mice fed with a control diet at the age of 24 weeks, endothelial dysfunction was present as evidenced by the loss of vasodilatation response and paradoxical vasoconstriction (Ach-induced response: − 9.7 ± 2.9% in BCA and − 25.7 ± 4.5% in LCA, Fig. 1). In PK-treated and in MK-treated ApoE/LDLR^−/−^ mice, Ach-induced responses reversed to vasodilation and achieved similar magnitude in BCA: 4.7 ± 4.0% and 8.0 ± 2.9%, respectively (Fig. 1a). For Ach-induced response in LCA in PK-treated and MK-treated ApoE/LDLR^−/−^ mice, the responses were also similar: 9.7 ± 3.9% and 7.5 ± 1.6%, respectively (Fig. 1b).

### PK and MK reversed TNF-induced endothelial dysfunction in the isolated mouse aorta in vitro

**Fig. 2:**
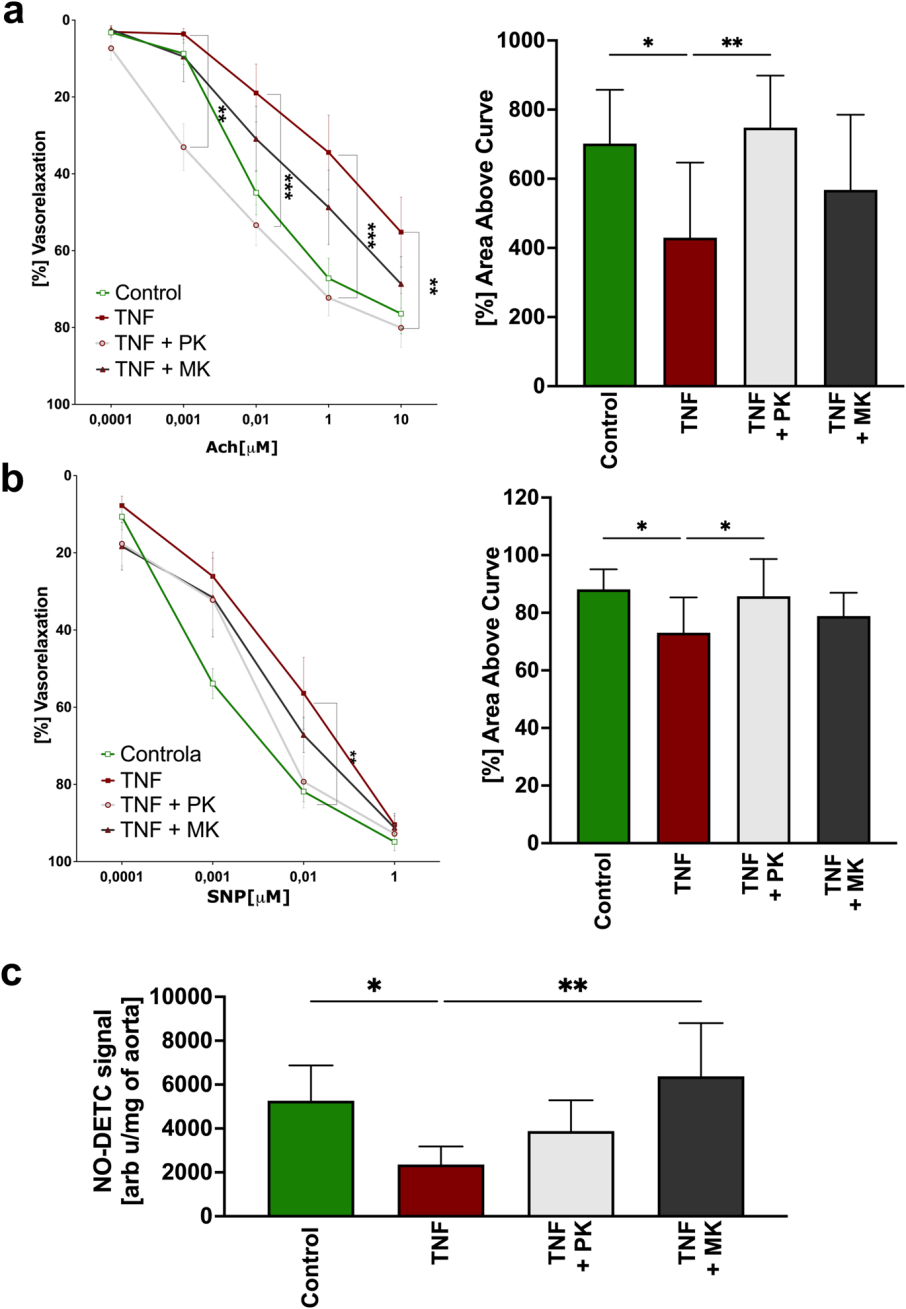
PK and MK improve endothelial function in the isolated aorta. Endothelium-dependent (**a**) and endothelium-independent (**b**) aortic dilatory responses were measured using a myograph after 24 h incubation with TNF in the presence or absence of PK (10 μM) or MK (10 μM). Subsequently in post-incubation aorta tissue, NO production was measured using EPR (**c**). Results are presented as mean ± SD *n* = 6–10. Statistical analysis was performed using one-way or two-way ANOVA with Dunnett’s multiple comparisons. The symbols *, **, and *** indicate statistical significance at *p* < 0.05, 0.01, and 0.001, respectively

Incubation of isolated aortic rings with TNF (10 ng/mL) for 24 h significantly impaired the Ach-induced, endothelium-dependent (Fig. 2a) and slightly impaired the SNP-induced, endothelium-independent (Fig. 2b) vasodilatation. MK (MK-7; 10 μM) tended to protect against the impairment of endothelium-dependent vasodilation induced by TNF (Fig. 2a), while PK (10 μM) afforded significant prevention of the TNF-induced impairment of endothelium-dependent vasodilation (Fig. 2a). PK and MK had a slight effect on the SNP-induced responses (Fig. 2b). The effect of PK and MK on endothelial function was also assayed by the measurements of NO production: PK and MK fully or partially respectively reversed TNF-induced impairment in NO production (Fig. 2c).

### PK and MK inhibited replicative senescence in vitro

**Fig. 3:**
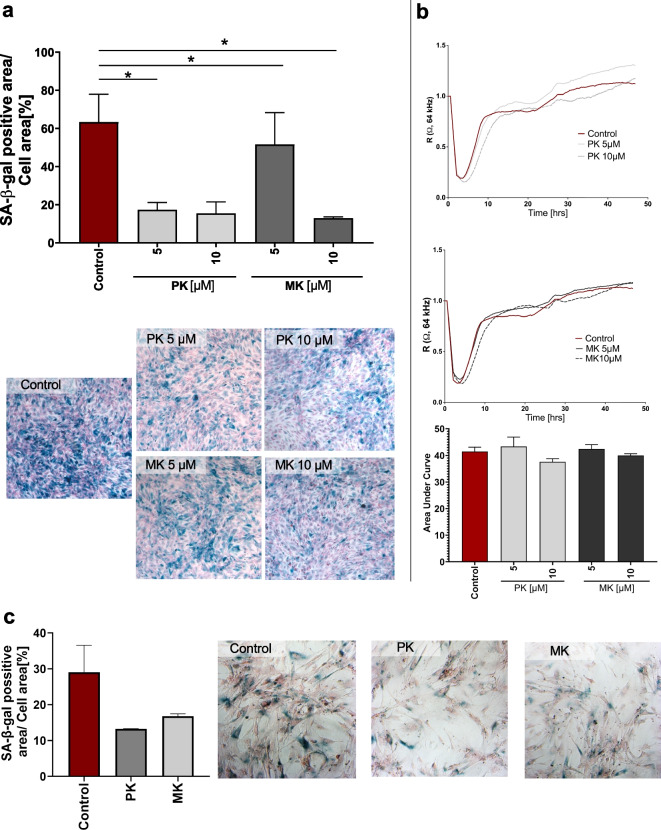
Anti-senescence activity of PK and MK on replicative senescence in endothelial and vascular smooth muscle cells. The inhibitory influence of PK and MK on replicative senescence in porcine aortic endothelial cells (PAEC) was assessed by measurement of SA-β-gal activity (**a**) and proliferation rate in wound healing assay (**b**) after 24 h of incubation with PK or MK (5 or 10 µM). The inhibitory influence of PK and MK on replicative senescence in human vascular smooth muscle cells (04/35F/11A) was assessed by measurement of SA-β-gal activity (**c**). Results are presented as mean ± SEM *n* = 3 (endothelial cells) *n* = 2 (vascular smooth muscle cells). Statistical analysis was performed using two-way ANOVA with Dunnett’s multiple comparisons test. The symbol * indicates statistical significance at *p* < 0.05

Next, we determined if there was an effect of PK and MK (MK-7) on cell senescence in vitro. PAECs at the 5th–7th passage displayed high senescence-associated β-galactosidase activity that was detected as a 60% SA-β-gal positive area of cells (Fig. 3a). Incubation of senescent PAEC with PK (5 μM) for 24 h decreased β-galactosidase activity, while the same concentration of MK did not exert a similar protective effect. Increasing the concentrations of PK and MK to 10 μM decreased β-galactosidase activity by a comparable degree, by approximately 70% (Fig. 8f). Incubation of senescent PAECs with PK or MK (5 μM or 10 μM) had no significant effect on cell proliferation in the wound healing assay (Fig. 3b). Similar to the findings obtained in the PAEC cells, PK and MK inhibited SA-β-gal activity in 04/35F/11 vascular smooth muscle cells in a model of replicative senescence (Fig. 3c).

## PK and MK inhibited stress-induced senescence in vitro

### X-ray radiation-induced senescence

**Fig. 4:**
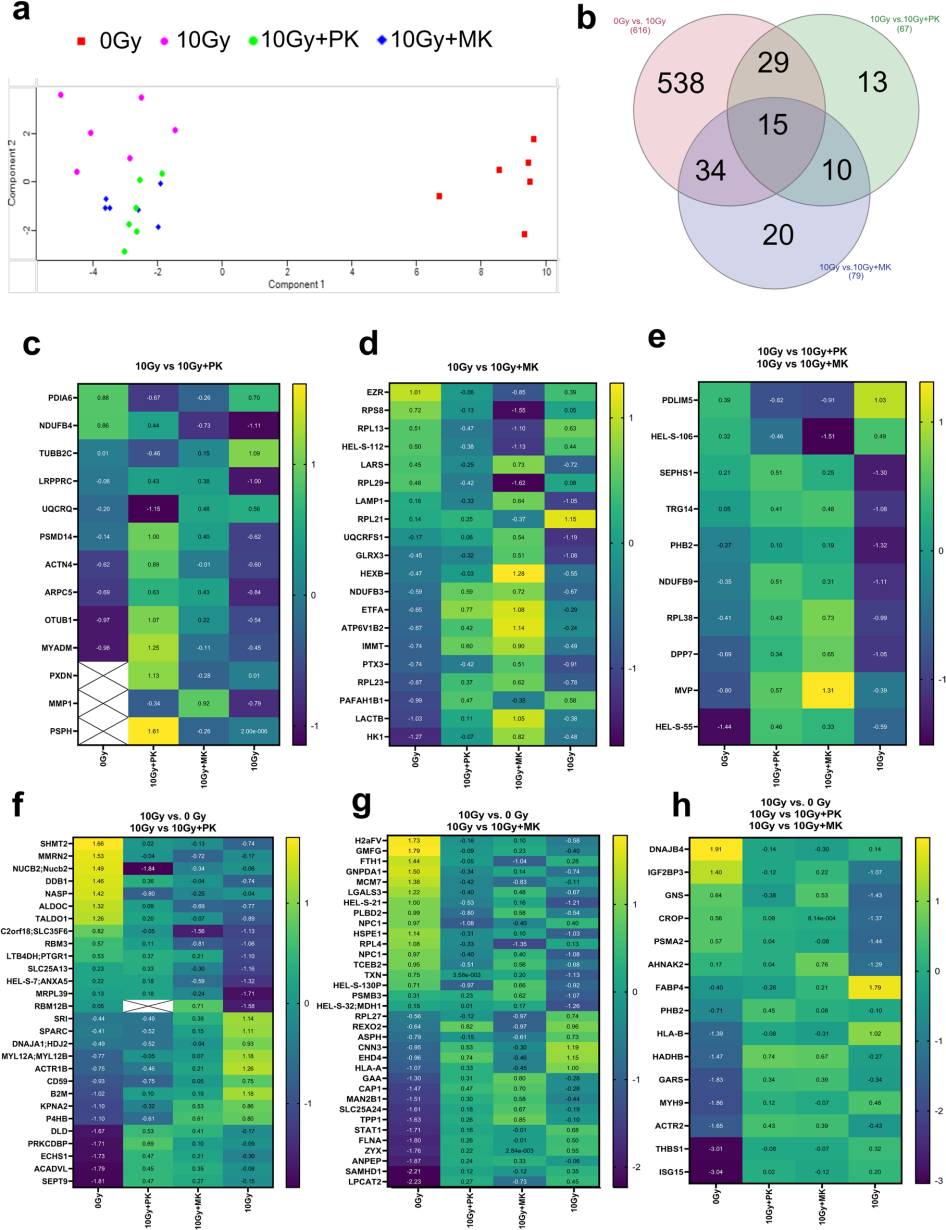
Anti-senescence activity of PK and MK on stress-induced senescence induced by X-ray radiation in human primary endothelial cells HAEC. The Principal Component Analysis (PCA) plot (**a**) demonstrates the variation between the studied groups 0 Gy (red), 10 Gy (magenta), 10 Gy + PK (green), and 10 Gy + MK (blue). Venn diagram (**b**) shows the number of differentially expressed proteins (DEP) in the following comparisons: 0 Gy vs. 10 Gy (616), 10 Gy vs. 10 Gy + PK (67), and 10 Gy vs. 10 Gy + MK (79). In the heat maps (**c**–**h**), the detailed DEP for the following comparisons has shown DEP differed in response to incubation with PK (**c**), MK (**d**), or either PK or MK compared to 10 Gy group (**e**). Moreover, the heat maps (**f**–**h**) demonstrate proteins that were altered in response to radiation (10 Gy) compared to the control group (0 Gy) and subsequently deregulated by the incubation with PK (**f**), MK (**g**), or PK as well as MK (**h**). The heat maps were generated using normalized Z-scored expression data and included only proteins for which the statistically significant differences have been determined in particular groups. Values in the cells mean a mean of *n* = 6. Statistical analysis was performed using Perseus software—MaxQuant

To understand the anti-senescence mechanism of PK and MK, proteomic analysis of HAEC in an X-ray radiation-induced senescence model was performed. The 3591 proteins have been identified in total in HAECs. After 10 days following irradiation—with or without PK or MK (MK-7) incubation—762 proteins were differentially expressed (DEP) 0 Gy vs. 10 Gy (616), 10 Gy vs. 10 Gy + PK (67), and 10 Gy vs. 10 Gy + MK (79) (Fig. 4a, b). As shown in a Venn diagram (Fig. 4b) and further presented on the heatmaps, 13 DEPs were exclusively affected by PK (10 Gy vs. 10 Gy + PK, Fig. 4c); 20 DEPs were exclusively regulated by MK (10 Gy vs. 10 Gy + MK, Fig. 4d) with 10 DEPs common for both vitamins (10 Gy vs. 10 Gy + PK and 10 Gy vs. 10 Gy + MK, Fig. 4e). Heatmaps (Fig. 4f–h) also show proteins that were commonly altered by irradiation and then their expression was decreased or increased in response to 10 days of incubation with PK (0 Gy vs. 10 Gy and 10 Gy vs. 10 Gy + PK—29 proteins, Fig. 4f), or with MK (0 Gy vs. 10 Gy and 10 Gy vs. 10 Gy + PK—34 proteins, Fig. 4g), or either by PK or MK (0 Gy vs. 10 Gy, 10 Gy vs. 10 Gy + PK, and 10 Gy vs. 10 Gy + MK—15 proteins, Fig. 4h).

We then focused on DEPs involved in cell senescence, oxidative stress, and response to DNA damage caused by radiation; there were changes in response to PK or MK. Incubation of HAEC with PK for 10 days after radiation significantly altered the expression of proteins involved in the following pathways: oxidative stress inhibition (TUBB2C [48], DNAJA1 [49]), DNA repair, including nucleotide excision and double strand breaks DNA repair (DDB1 [50], ACTN4 [51], OTUB1 [52], RBM3 [53], SPARC [54], DNAJA1 [49] KPNA2 [55],), and senescence (PSMS14 [56], PXDN [57], MMP1 [58], PSPH [59], ANXA5 [60], CD59 [61], B2M [62]). The 10-day incubation of HAEC with MK after radiation significantly affected the following pathways: DNA repair including mismatch repair (HEXB [63], LACTB [64], H2AFV [65], HLA-A [66], SLC25A24 [67], TPP1 [68], SAMHD1 [69]) and senescence (LAMP-1 [70], GLRX3 [71], HK1 [72], GMFG [73], FTH1 [74], MCM7 [75], LGALS3 [76], TXN [77], ASPH [78], TPP1 [68], STAT1 [79], FLNA [80]). The proteins that were significantly altered in response to incubation either with PK as MK included proteins involved in oxidative stress regulation (MYH9 [81]), response to DNA damage (HLA-B [66], HADHB [82], ISG15 [83]), and senescence (DNAJB4 [84], AHNAK [85], IGF2BP3 [86], FAB4 [87], GARS [88], ISG15 [89], SEPHS [90], MVP [91]). Among the listed processes directly related to senescence, it is worth mentioning the positive impact of PK and MK addition on the cellular response to cytokines (e.g., THBS1, PMS, KPNA, MX1, RBMX, MAP3K), response to interferon-gamma (e.g., B2N, SNCA, ICAM1, PML, STAT1), extracellular matrix organization (e.g., ITGB4, LGALS3, CTSD, LAMB1), increased mitochondrial fatty acid β oxidation (DECR1, HSD17B4, HADHA, ECHS1, ACADVL, HADHB), Krebs cycle (MDH2, DLD, IDH3B, ACO2, PCK2, FH, IDH3A, HEL60, MDH1), respiratory electron transport (e.g., DLD, NDUFA4, NDUFA6, ETFA, ATP5J), and tRNA aminoacylation (e.g., GARS, YARS, DARS, LARS, NARS, AARS, KARS).

**Fig. 5:**
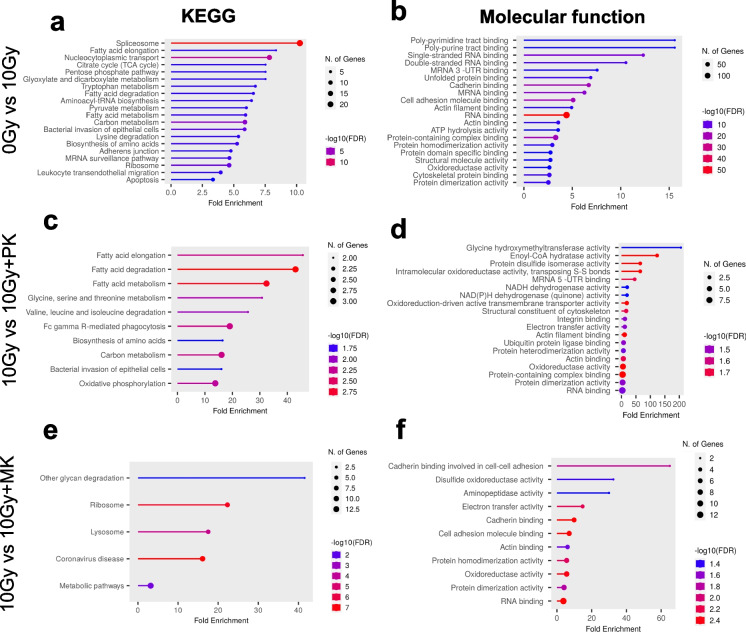
Proteomics analysis of KEGG and molecular processes in human primary endothelial cells HAEC irradiated using X-ray incubated with PK or MK. Histograms demonstrate the statistically significant enrichment of DEPs in response to irradiation 0 Gy vs 10 Gy (**a**–**b**) and subsequent incubation with PK—10 Gy vs 10 Gy + PK (**c**–**d**) or MK—10 Gy vs 10 Gy + MK (**e–**f) for KEGG (**a**, **c**, **e**) and molecular function (**b**, **d**, **f**) pathways

Additionally, general gene ontology–based proteomic analysis of KEGG (Fig. 5a, c, e) and molecular function of DEPs (Fig. 5b, d, f) in HAEC cells, at 10 days after irradiation (0 Gy vs. 10 Gy), indicated changes in multiple processes in response to radiation, including translation, synthesis, and protein folding, adhesion, cytoskeleton reorganization, and energy metabolism (Fig. 5a, b). When cells were incubated 10 days after radiation with PK (10 Gy vs. 10 Gy + PK), the expression of proteins involved in folding, binding, and protein activity, fatty acid and amino acid metabolism, energy metabolism, mitochondrial function, cytoskeleton organization, junctions, adhesion, and the immune response were altered (Fig. 5c, d). In response to a 10-day incubation of irradiated HAEC with MK, deregulated proteins included proteins regulating cell adhesion, electron transfer, cytoskeleton organization, and RNA binding. (Fig. 5e, f). Both PK and MK had an influence on several molecular processes including electron transfer activity, oxidoreductase activity, protein dimerization activity, actin binding, and RNA binding.

All data obtained by proteomic LC–MS-based analysis, which has been visualized on graphs, are enclosed as a table in Supplementary Table 1.

### Prelamin A overexpression-induced senescence

**Fig. 6:**
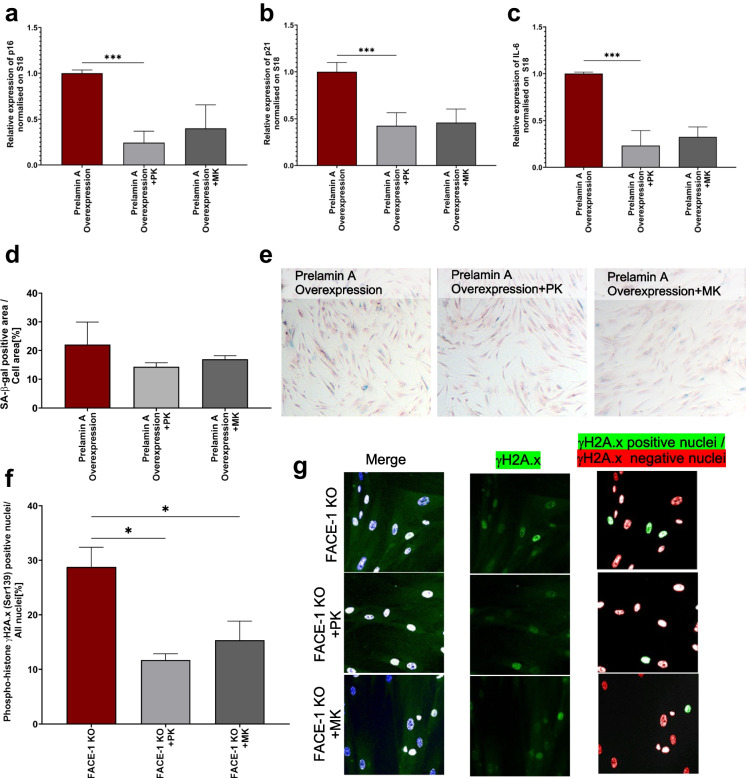
Anti-senescence activity of PK and MK on stress-induced senescence induced by prelamin A accumulation in human primary vascular smooth muscle cells 04/35F/11A. The effects of 48 h incubation with PK and MK (10 µM) on senescence in cells with prelamin A overexpression were assessed by changes in the gene expression of senescence-associated genes p16 (**a**), p21 (**b**), IL-6 (**c**), and SA-β-gal activity (**d**, **e**). To define the mechanism of anti-senescence activity of PK and MK (10 µM), the DNA damage was assessed based on ratio of phosphorylated histone γH2A.X (Ser139) positive nuclei to all nuclei (phosphorylated histone γH2A.X (Ser139) positive nuclei + phosphorylated histone γH2A.X (Ser139) negative nuclei) and was measured in the cells with FACE-1 knockout (resulting in prelamin A accumulation) (**f**–**g**). Results are presented as mean ± SEM *n* = 3. The symbols * and *** indicate statistical significance at *p* < 0.05 and 0.001, respectively

Given that prelamin A accumulation disrupts the DNA repair pathways, contributing to increased accumulation of DNA damage, prelamin A accumulation results in stress-induced premature senescence [92]. Therefore, the effect of PK and MK on senescence induced in 04/35F/11A cells by prelamin A overexpression was studied. A 48-h incubation of cells with PK or MK (MK-7; 10 μM) significantly decreased gene expression of senescence-associated genes p16^INK4a^ (Fig. 6a), p21 (Fig. 6b), and Il-6 (Fig. 6c). There was however no significant effect of PK and MK (MK-7) on the activity of SA-β-gal (Fig. 6d, e). In the model of FACE-1 knockout cells (which also induce prelamin A accumulation), PK as well as MK (MK-7) decreased phosphorylation of γH2A.x expression and prevented the associated DNA damage (Fig. 6f, g).

## PK and MK inhibited TNF-induced inflammation in HAEC

**Fig. 7:**
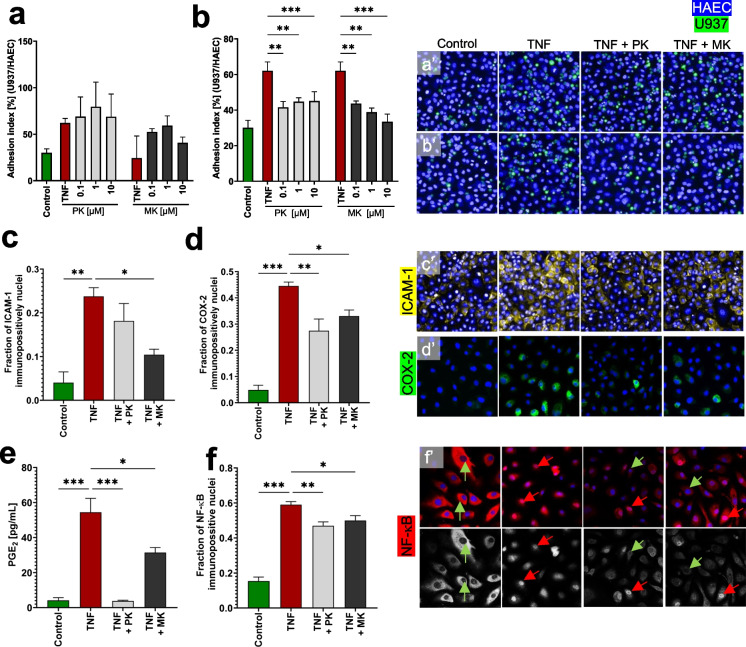
Anti-inflammatory activity of PK and MK in human primary endothelial cells HAEC. The anti-inflammatory effects of PK and MK (10 µM) on TNF (10 mg/mL, 24 h) pro-inflammation-stimulated HAEC were assessed by analyzing monocyte (U937) adhesion to pro-inflammation-activated endothelium when the U937 (a, a′) or HAEC (b, b′) where pre-incubated 24 h with PK or MK before TNF, and then subsequently incubated 24 h with TNF together with PK or MK. Additionally, protein expression of ICAM-1 (c, c′) and COX-2 (d, d′) and PGE_2_ production (e) was measured. To explain the mechanism of anti-inflammatory activity of PK and MK, the NF-κB nuclear translocation was analyzed immunocytochemically (f, f′). The black and white images demonstrate NF-κB staining (Cy3 channel separately), red arrows indicate NF-κB positive nuclei, while green arrows indicate NF-κB negative nuclei. Results are presented as mean ± SEM *n* = 3. Statistical analysis was performed using one-way ANOVA with Dunnett’s multiple comparisons test for the pro-inflammatory stimulated control. The symbols *, **, and *** indicate statistical significance at p < 0.05, 0.01, and 0.001, respectively

To study the effect of PK and MK (MK-7) on endothelial inflammation, the adhesion of monocytes to activated endothelium as well as expression of ICAM-1, COX-2, NFκB, and PGE_2_ production was analyzed. Pre-treatment of the human promyelocytic cells U397 with PK or MK did not affect the cell adhesion to TNF-activated HAECs (Fig. 7a and a′). In contrast, pre-treatment of HAEC with either PK or MK before contact with monocytes inhibited the adhesion of U397 cells to endothelial cells (Fig. 7b and b′). PK and MK were equipotent and were effective in the concentration range of 0.1–10 μM. TNF stimulation increased the expression of the adhesion protein ICAM-1, and treatment with 10 μM PK or MK inhibited ICAM-1 expression (Fig. 7c and c′).

Similarly, both PK and MK (MK-7) decreased the fraction of COX-2 immunopositive HAEC in TNF-activated endothelium (Fig. 7d and d′). TNF-induced PGE_2_ production was also inhibited by PK and by MK (Fig. 7e).

The inhibitory effect of PK and MK (MK-7) on inflammatory markers was consistent with the effects on the activation of the NF-κB pathway, and the fraction of NF-κB immunopositive nuclei was slightly but significantly decreased in response to treatment with either PK or MK (Fig. 7f and f′).

**Fig. 8:**
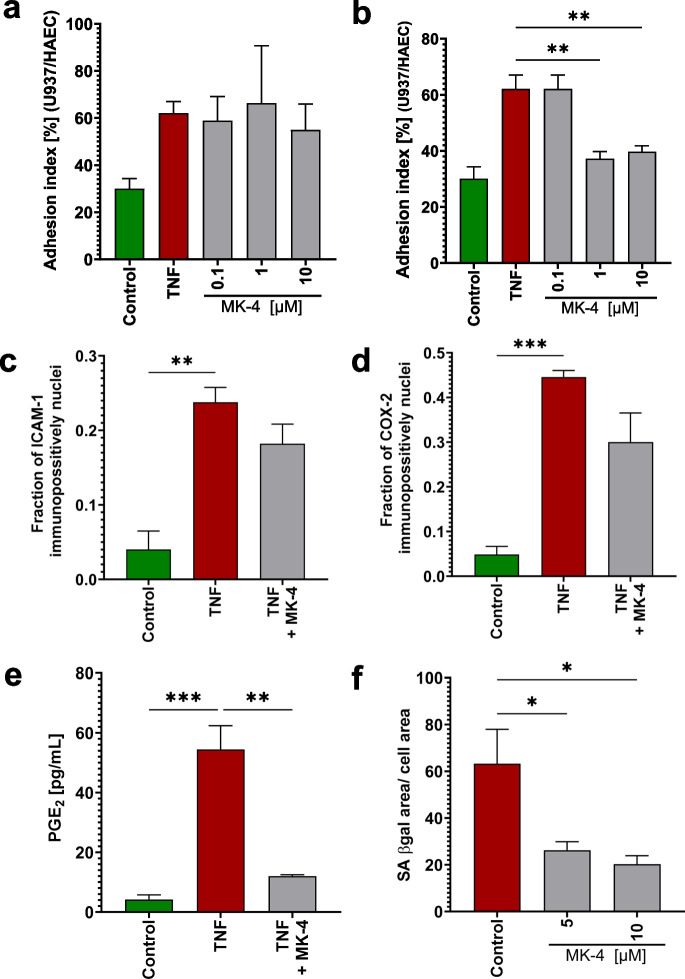
Anti-inflammatory and anti-senescence effects of another member of the MK group—MK-4. The results of anti-inflammatory and anti-senescence effects obtained for MK-7 have been verified for another representant MK group—MK-4, for selected markers. The anti-inflammatory effects of MK-4 (10 µM) on TNF (10 mg/mL, 24 h) pro-inflammation-stimulated HAEC were assessed by analyzing monocyte (U937) adhesion to pro-inflammation-activated endothelium when the U937 (**a**) or HAEC (**b**) where pre-incubated 24 h before TNF with MK-4 and incubated 24 h with TNF together with MK-4. Moreover, protein expression of ICAM-1 (**c**) COX-2 (**d**) and PGE_2_ production (**e**) was measured. To assess the anti-senescence activity of MK-4, the changes of SA-β-gal activity in response to 24 h incubation with 5 μM or 10 μM MK-4 were measured in PAEC (**f**). Results are presented as mean ± SD *n* = 3. Statistical analysis was performed using one-way ANOVA with Dunnett’s multiple comparisons test for the pro-inflammatory stimulated control. The symbols *, **, and *** indicate statistical significance at *p* < 0.05, 0.01, and 0.001, respectively

The effects of MK-4 were comparable to the effect of MK-7 for all tested parameters: adhesion of monocytes (Fig. 8a, b), ICAM-1 (Fig. 8c), COX-2 (Fig. 8d) expression, and PGE_2_ production (Fig. 8e).

## Vascular uptake of PK and conversion to MK-4 in isolated aorta, HAEC, and MOVAS

**Fig. 9:**
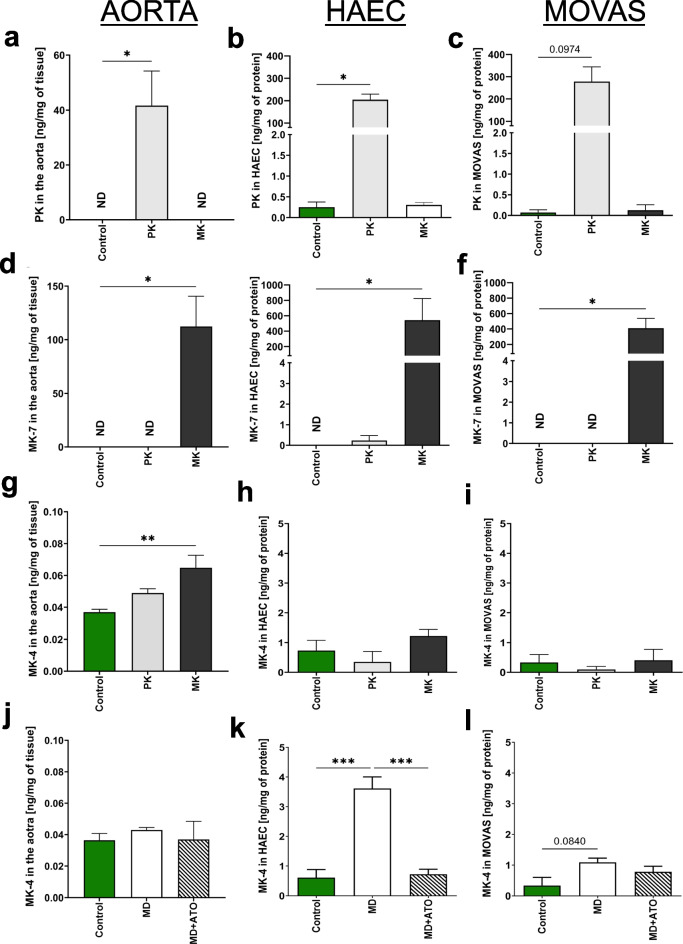
The PK and MK uptake and synthesis of endogenous MK-4 in isolated aorta, HAEC, and MOVAS. The uptake capacity of exogenous PK (**a**–**c**) and MK (**d**–**f**), endogenous production of MK-4 form PK (**g**–**i**) and MD, and the influence of atorvastatin (ATO, 1 μM) on inhibition of MD prenylation (**j–**l) were assessed in the aorta (**a**, **d**, **g**, **j**), HAEC (**b**, **e**, **h**, **k**), and MOVAS (**c**, **f**, **i**, **l**) after 24 h of incubation with PK and MK or MD (5 μM for aorta or 1 μM for cells). Results are presented as mean ± SD *n* = 3. Statistical analysis was performed using one-way ANOVA with Dunnett’s multiple comparisons test. The symbols ** and *** indicate statistical significance at *p* < 0.01 and 0.001, respectively. ND not detected

To verify whether PK could be uptaken and metabolized to MK-4, PK (Fig. 9a–c) or MK (Fig. 9d–f) were added to the medium and incubated with isolated aorta (Fig. 9a, d), HAEC (Fig. 9b, e), or MOVAS cells (Fig. 9c, f). PK and MK were both taken up by the cells and aorta tissue, suggesting that PK, as well as MK, can be uptaken by vessels when available as substrates for vitamin K–dependent processes. Incubation of HAEC, MOVAS, or aortic tissue with PK (5 µM) or MK (MK-7; 5 µM) also increased the concentration of MK-4, suggesting that direct uptake of PK and the vascular conversion of PK to MK-4 both processes occurred (Fig. 9g–i).

Incubation of HAEC with MD (1 µM) significantly increased intracellular MK-4 levels, an effect, which was inhibited by atorvastatin (ATO, 1 μM), an inhibitor of prenylation mechanisms [19], and thereby of MK-4 synthesis (Fig. 9k, h). In MOVAS, these effects were less pronounced. In the aorta, MD did not increase MK-4 levels (Fig. 9j).

## Discussion

For many years, MK has been considered as a cardioprotective and vascular protective substance due to its ability to inhibit the calcification of blood vessels and cardiac valves. However, recent data indicate that the beneficial activity of MK in the vessel wall can also involve additional mechanisms, including beneficial effect on endothelial function, that was not related to the vascular calcification process [23]. On the other hand, vasoprotective effects of PK were suggested based on the finding that low PK plasma levels were associated with increased cardiovascular risk [93, 94]. Yet, a number of studies in humans did not find any beneficial vascular effects of PK [25, 95, 96].

The present study demonstrated the beneficial effects of both PK and MK on endothelial function, cellular senescence, and vascular inflammation. Based on these studies, we also conclude that PK- and MK-afforded vasoprotective effects can be ascribed, at least in part, to their anti-senescence activity (at least in part via decreasing DNA damage) and to their anti-inflammatory activity (at least in part via inhibition of NF-κB activation). Given the uptake of PK by vascular wall and the generation of endogenous MK-4 in the aorta incubated with PK, we claim that the vasoprotective effects of PK can be mediated—at least in part—by its active metabolite—MK-4.

In the present study, we demonstrated a comparable effect of exogenous PK and MK to improve endothelial dysfunction in ApoE/LDLR^−/−^ mice. We used ApoE/LDLR^−/−^ mice which constitute a well-characterized model of systemic endothelial dysfunction [46], and atherogenesis that is however not associated with cardiac dysfunction even in mice at the age of 4–6 months, due to compensatory vascular and metabolic mechanisms [97–99]. In our previous study using an MRI-based approach to assess endothelial function in vivo, we demonstrated that MK-7 improved endothelial function in this model [25]. Here, we investigated the effects of PK or MK-enriched diet on endothelial function in BCA and LCA using a similar experimental approach. We used a high dose of MK-4 or PK for the same period of 8 weeks to compare with the previous study. In our previous work [25], we observed a similar degree of improvement in endothelium-dependent vasodilation and increase in plasma nitrate concentration induced by a low (0.03 mg/kg b.w./day) or high (10 mg/kg b.w./day) dose of MK-7, even though there was a dose-dependent increase in plasma MK-7 and MK-4 concentrations after 8 weeks of dietary supplementation with MK-7 in ApoE/LDLR^−/−^. Given the different bioavailabilities of PK and MK, here, to exclude that the too-low dose could be the reason for the weaker activity of PK or MK if detected, we used a high dose of 10 mg/kg for both PK and MK and demonstrated to our knowledge, for the first time, their similar profile of vascular action of PK and MK-4 involving improvement of endothelial function in vivo that was comparable to previously demonstrated effect of MK-7 on endothelial function in the same experimental model.

In clinical trials, vitamin K was used in various doses including also such high doses of PK as 10 mg/day [12] or even 45 mg/day of MK-4 [100]. In this context, our high dose of vitamin K stays in a similar range after adjustments to the faster metabolic rate of mice vs humans. Furthermore, much higher doses (50 mg/kg/day) have been used in rodents, with positive therapeutic effects [101].

Vascular dysfunction is often mechanistically related to cellular senescence. Indeed, accumulation of senescent cells and senescence-associated secretory (pro-inflammatory) phenotype of cells is considered a risk factor during the development of cardiovascular disease associated with aging [102–106]. Increasing attention is being paid to a new concept of early (premature) vascular aging (EVA) that could accelerate cardiovascular diseases. In fact, EVA with a more rapid course of characteristic age-related changes in individuals as compared to others with the same biological age resulted in accelerated vascular inflammation and oxidative stress–associated arterial stiffness [107]. Knowing that both age-related vascular aging and EVA represent significant cardiovascular risk factors that could be mechanistically related to cellular senescence, we studied the anti-senescence activity of PK and MK. Of note, ApoE and inflammation contribute to the regulation of cell senescence [108–110]. Considering that many factors can induce premature senescence, we have studied the anti-senescence effects of MK or PK not only in models of replicative senescence but also in stress-induced senescence. In all senescence models tested, the beneficial activity of PK and MK was comparable.

Given that oxidative stress and associated DNA damage are considered an important factor in aging [111–114] and vitamin K is considered a strong antioxidant [115, 116], a logical next step in our studies was to test if MK or PK can ameliorate DNA damage in senescent cells. We used cellular models of VSMC—where the DNA damage has been induced by the prelamin A accumulation [42]. Our results showed that both PK and MK attenuated the expression of phosphorylated γH2A.x associated with DNA damage. Accordingly, both PK and MK may act as inhibitors of DNA damage, most likely through antioxidant effects, but other mechanisms could also be involved. Interestingly, both PK and MK regulated multiple proteins associated with senescence and response to DNA damage/DNA repair in the X-ray-induced senescence in HAEC endothelial cells. In the literature, the only available information regarding the effect of vitamin K on DNA damage relates to MD. Importantly, these studies—which utilize high concentrations of MD—showed toxic effects including the induction of DNA damage [117]. In stark contrast to these data, the present results demonstrated for the first time the vascular protective role of vitamin K that could be at least partially ascribed to the prevention of DNA damage. These results may provide a starting point for further studies, to verify whether the DNA-protective mechanism of vitamin K may be mechanistically related to the presence of prenyl side chains rather than to naphthoquinone moiety.

In the context of the potential beneficial role of vitamin K in age-related diseases, the literature primarily focuses on neurodegenerative diseases [118, 119]. The anti-senescence effects of PK and MK presented in the current report expand their possible therapeutic on various forms of aging-associated cardiovascular diseases.

Cardiovascular diseases are closely associated with vascular inflammation [120–124]. For instance, vascular infiltration of monocytes is considered one of the culprits of the early phases of atherogenesis, followed by the formation of foam cells, consequently contributing to the progression of atherosclerosis [124]. Moreover, several studies show that high levels of adhesion molecules and activation of extracellular matrix metalloproteinases in vascular tissue itself correlate with increased cardiovascular risk [125–130]. Inflammation is also inextricably linked to aging and accompanies age-related cardiovascular diseases. Initially, the anti-inflammatory effects of MK were attributed to its ability to modulate immune cell activation [13, 14]. Subsequent studies demonstrated that the action of MK, PK, or MD can also involve effects on other cell types such as fibroblasts and osteocytes [131, 132]. The present study demonstrated the ability of PK and MK to regulate the expression of adhesion proteins and to inhibit the process of monocyte adhesion to activated endothelial cells. Additionally, our data demonstrated that both PK and MK inhibited COX-2 expression and PGE_2_ production by activated endothelial cells, which is in accordance with previous data obtained in macrophages [14]. Finally, the results of our study suggest that the mechanism of MK’s and PK’s anti-inflammatory activity may be, at least in part, related to the inhibition of NF-κB activation [13, 14].

The important part of the presented results related to the characterization of PK uptake by isolated aorta, as well as cultured endothelial or smooth muscle cells. We demonstrated that incubation of isolated aorta, endothelial cells, or smooth muscle cells with PK resulted in a substantial increase in intracellular levels of PK in the aorta tissue or cultured vascular cells. PK uptake was however not associated with a significant increase in MK-4 contents in vascular cells but was associated with a modest increase in MK-4 content in the aorta. On the other hand, MD induced a robust MK-4 production in cultured vascular cells that was inhibited by atorvastatin, an inhibitor of MK-4 synthesis. These results demonstrated robust uptake of PK into cultured vascular cells as well as isolated aorta and a possible metabolism of PK to MK-4 in the vascular wall. However, systemic conversion of PK to MK-4 may also be of importance in this context. Further studies in vivo are needed to explore the metabolism of PK to MK-4 in the context of vascular function in more detail to delineate the cell types and enzymatic steps involved in these processes. In fact, in an in vivo setting, the vascular delivery of dietary PK is subjected to many factors such as storage in the liver [133, 134], lipoprotein-dependent transport [135], conversion in intestine [23, 24], and hydrophobic character [136] that could not be mimicked in isolated cells or isolated vessel setting. All of these factors may contribute to PK distribution and metabolism and determine PK and MK-4 content in the vasculature that will translate into vasoprotective action. Nevertheless, based on our results, we claim that PK and MK both may support vascular homeostasis and their relative roles might be related to their availability as substrates for vitamin K–dependent processes in the vascular wall.

In conclusion, the results presented in the current study expand the existing knowledge regarding the vascular protective activity of vitamin K and suggest that not only MK but also PK may exert vascular protective roles, including protection of endothelial function, inhibition of cellular senescence, suppression of vascular inflammation, and inhibition of DNA damage. These effects may be, at least in part, due to PK itself or may be related to PK vascular metabolism to MK-4. The pharmacological activities of exogenous PK—the major dietary source of vitamin K—described in the present work should stimulate further preclinical, and, eventually, clinical studies focused on the supplementation of PK for cardiovascular diseases, age-related diseases, and vascular inflammation. The documented relationship between PK plasma level and cardiovascular risk [93, 94] together with our results demonstrating vasoprotective action of PK suggests that dietary PK emerges as an important nutritional protective factor against the development of vascular dysfunction and aging of the cardiovascular system.

### Supplementary Information

Below is the link to the electronic supplementary material.

Supplementary file1 (DOCX 23 KB)

Supplementary Figure 1  The effect transfection of the 04/35F/11A vascular smooth muscle cells with the scrambled control siRNA vs FACE-1 siRNA on phosphorylated yH2A.x expression (PDF 241 KB)

Supplementary file3 (XLSX 2488 KB)

## Data Availability

The data that support the findings of this paper are available from the corresponding author, upon reasonable request.

## References

[1] Sato T, Schurgers LJ, Uenishi K. Comparison of menaquinone-4 and menaquinone-7 bioavailability in healthy women. Nutr J. 2012;12(11):93. 10.1186/1475-2891-11-93. 10.1186/1475-2891-11-93 PMC3502319 23140417

[2] Beulens JW, Booth SL, van den Heuvel EG, Stoecklin E, Baka A, Vermeer C. The role of menaquinones (vitamin K_2_) in human health. Br J Nutr. 2013;110(8):1357–68. 10.1017/S0007114513001013. 23590754 10.1017/S0007114513001013

[3] Booth SL. Roles for vitamin K beyond coagulation. Annu Rev Nutr. 2009;29:89–110. 10.1146/annurev-nutr-080508-141217. 19400704 10.1146/annurev-nutr-080508-141217

[4] Willems BA, Vermeer C, Reutelingsperger CP, Schurgers LJ. The realm of vitamin K dependent proteins: shifting from coagulation toward calcification. Mol Nutr Food Res. 2014;58(8):1620–35. 10.1002/mnfr.201300743. 24668744 10.1002/mnfr.201300743

[5] van den Heuvel EG, van Schoor NM, Lips P, Magdeleyns EJ, Deeg DJ, Vermeer C, den Heijer M. Circulating uncarboxylated matrix Gla protein, a marker of vitamin K status, as a risk factor of cardiovascular disease. Maturitas. 2014;77(2):137–41. 10.1016/j.maturitas.2013.10.008. 24210635 10.1016/j.maturitas.2013.10.008

[6] Chatron N, Hammed A, Benoît E, Lattard V. Structural insights into phylloquinone (vitamin K1), menaquinone (MK4, MK7), and menadione (vitamin K3) binding to VKORC1. Nutrients. 2019;11(1):67. 10.3390/nu11010067. 30609653 10.3390/nu11010067 PMC6357001

[7] Ichikawa T, Horie-Inoue K, Ikeda K, Blumberg B, Inoue S. Steroid and xenobiotic receptor SXR mediates vitamin K2-activated transcription of extracellular matrix-related genes and collagen accumulation in osteoblastic cells. J Biol Chem. 2006;281(25):16927–34. 10.1074/jbc.M600896200. 16606623 10.1074/jbc.M600896200

[8] Ohsaki Y, Shirakawa H, Miura A, Giriwono PE, Sato S, Ohashi A, Iribe M, Goto T, Komai M. Vitamin K suppresses the lipopolysaccharide-induced expression of inflammatory cytokines in cultured macrophage-like cells via the inhibition of the activation of nuclear factor κB through the repression of IKKα/β phosphorylation. J Nutr Biochem. 2010;21(11):1120–6. 10.1016/j.jnutbio.2009.09.011. 20149620 10.1016/j.jnutbio.2009.09.011

[9] Vos M, Esposito G, Edirisinghe JN, Vilain S, Haddad DM, Slabbaert JR, Van Meensel S, Schaap O, De Strooper B, Meganathan R, Morais VA, Verstreken P. Vitamin K2 is a mitochondrial electron carrier that rescues pink1 deficiency. Science. 2012;336(6086):1306–10. 10.1126/science.1218632. 22582012 10.1126/science.1218632

[10] Tirapelli CR, Mingatto FE, de Oliveira AM. Vitamin K(1) prevents the effect of hypoxia on phenylephrine-induced contraction in the carotid artery. Pharmacology. 2002;66(1):36–43. 10.1159/000063255. 12169764 10.1159/000063255

[11] Tasatargil A, Cadir B, Dalaklioglu S, Yurdakonar E, Caglar S, Turkay C. Effects of vitamin K1 supplementation on vascular responsiveness and oxidative stress in a rat femoral osteotomy model. Cell Biochem Funct. 2007;25(5):485–90. 10.1002/cbf.1335. 16929463 10.1002/cbf.1335

[12] Kolahi S, Pourghassem Gargari B, Mesgari Abbasi M, Asghari Jafarabadi M, Ghamarzad Shishavan N. Effects of phylloquinone supplementation on lipid profile in women with rheumatoid arthritis: a double blind placebo controlled study. Nutr Res Pract. 2015;9(2):186–91. 10.4162/nrp.2015.9.2.186. 25861426 10.4162/nrp.2015.9.2.186 PMC4388951

[13] Pan MH, Maresz K, Lee PS, Wu JC, Ho CT, Popko J, Mehta DS, Stohs SJ, Badmaev V. Inhibition of TNF-α, IL-1α, and IL-1β by pretreatment of human monocyte-derived macrophages with menaquinone-7 and cell activation with TLR agonists in vitro. J Med Food. 2016;19(7):663–9. 10.1089/jmf.2016.0030. 27200471 10.1089/jmf.2016.0030

[14] Kieronska-Rudek A, Kij A, Kaczara P, Tworzydlo A, Napiorkowski M, Sidoryk K, Chlopicki S. Exogenous vitamins K exert anti-inflammatory effects dissociated from their role as substrates for synthesis of endogenous MK-4 in murine macrophages cell line. Cells. 2021;10(7):1571. 10.3390/cells10071571. 34206530 10.3390/cells10071571 PMC8303864

[15] Upadhyay A, Fontes FL, Gonzalez-Juarrero M, McNeil MR, Crans DC, Jackson M, Crick DC. Partial saturation of menaquinone in Mycobacterium tuberculosis: function and essentiality of a novel reductase. Men J ACS Cent Sci. 2015;1(6):292–302. 10.1021/acscentsci.5b00212. 10.1021/acscentsci.5b00212 PMC4582327 26436137

[16] Koehn JT, Crick DC, Crans DC. Synthesis and characterization of partially and fully saturated menaquinone derivatives. ACS Omega. 2018;3(11):14889–901. 10.1021/acsomega.8b02620. 31458155 10.1021/acsomega.8b02620 PMC6643618

[17] Cenci U, Qiu H, Pillonel T, Cardol P, Remacle C, Colleoni C, Kadouche D, Chabi M, Greub G, Bhattacharya D, Ball SG. Host-pathogen biotic interactions shaped vitamin K metabolism in Archaeplastida. Sci Rep. 2018;8(1):15243. 10.1038/s41598-018-33663-w. 30323231 10.1038/s41598-018-33663-w PMC6189191

[18] Conly JM, Stein K. The production of menaquinones (vitamin K2) by intestinal bacteria and their role in maintaining coagulation homeostasis. Prog Food Nutr Sci. 1992;16(4):307–43. 1492156

[19] Harshman SG, Shea MK, Fu X, Grusak MA, Smith D, Lamon-Fava S, Kuliopulos A, Greenberg A, Booth SL. Atorvastatin decreases renal menaquinone-4 formation in C57BL/6 male mice. J Nutr. 2019;149(3):416–21. 10.1093/jn/nxy290. 30753659 10.1093/jn/nxy290 PMC6398385

[20] Okano T, Shimomura Y, Yamane M, Suhara Y, Kamao M, Sugiura M, Nakagawa K. Conversion of phylloquinone (vitamin K1) into menaquinone-4 (Vitamin K2) in mice: two possible routes for menaquinone-4 accumulation in cerebra of mice. J Biol Chem. 2008;283(17):11270–9. 10.1074/jbc.M702971200. 18083713 10.1074/jbc.M702971200

[21] Thijssen HH, Vervoort LM, Schurgers LJ, Shearer MJ. Menadione is a metabolite of oral vitamin K. Br J Nutr. 2006;95(2):260–6. 10.1079/bjn20051630. 16469140 10.1079/bjn20051630

[22] Hirota Y, Tsugawa N, Nakagawa K, Suhara Y, Tanaka K, Uchino Y, Takeuchi A, Sawada N, Kamao M, Wada A, Okitsu T, Okano T. Menadione (vitamin K3) is a catabolic product of oral phylloquinone (vitamin K1) in the intestine and a circulating precursor of tissue menaquinone-4 (vitamin K2) in rats. J Biol Chem. 2013;288(46):33071–80. 10.1074/jbc.M113.477356. 24085302 10.1074/jbc.M113.477356 PMC3829156

[23] Ronden JE, Drittij-Reijnders MJ, Vermeer C, Thijssen HH. Intestinal flora is not an intermediate in the phylloquinone-menaquinone-4 conversion in the rat. Biochim Biophys Acta. 1998;1379(1):69–75. 10.1016/s0304-4165(97)00089-5. 9468334 10.1016/s0304-4165(97)00089-5

[24] Hegarty JM, Yang H, Chi NC. UBIAD1-mediated vitamin K2 synthesis is required for vascular endothelial cell survival and development. Development. 2013;140(8):1713–9. 10.1242/dev.093112. 23533172 10.1242/dev.093112 PMC3621489

[25] Bar A, Kus K, Manterys A, Proniewski B, Sternak M, Przyborowski K, Moorlag M, Sitek B, Marczyk B, Jasztal A, Skórka T, Franczyk-Żarów M, Kostogrys RB, Chlopicki S. Vitamin K2-MK-7 improves nitric oxide-dependent endothelial function in ApoE/LDLR−/− mice. Vascul Pharmacol. Vascul Pharmacol. 2019;122–123:106581. 10.1016/j.vph.2019.106581. 31421222 10.1016/j.vph.2019.106581

[26] Juanola-Falgarona M, Salas-Salvadó J, Martínez-González MÁ, Corella D, Estruch R, Ros E, Fitó M, Arós F, Gómez-Gracia E, Fiol M, Lapetra J, Basora J, Lamuela-Raventós RM, Serra-Majem L, Pintó X, Muñoz MÁ, Ruiz-Gutiérrez V, Fernández-Ballart J, Bulló M. Dietary intake of vitamin K is inversely associated with mortality risk. J Nutr. 2014;144(5):743–50. 10.3945/jn.113.187740. Erratum in: J Nutr. 2016 Mar;146(3):653. 10.3945/jn.113.187740 24647393

[27] Geleijnse JM, Vermeer C, Grobbee DE, Schurgers LJ, Knapen MH, van der Meer IM, Hofman A, Witteman JC. Dietary intake of menaquinone is associated with a reduced risk of coronary heart disease: The Rotterdam Study. J Nutr. 2004;134(11):3100–5. 10.1093/jn/134.11.3100. 15514282 10.1093/jn/134.11.3100

[28] Kostogrys RB, Franczyk-Zarow M, Gasior-Glogowska M, Kus E, Jasztal A, Wrobel TP, Baranska M, Czyzynska-Cichon I, Drahun A, Manterys A, Chlopicki S. Anti-atherosclerotic effects of pravastatin in brachiocephalic artery in comparison with en face aorta and aortic roots in ApoE/LDLR^−/−^ mice. Pharmacol Rep. 2017;69(1):112–8. 10.1016/j.pharep.2016.09.014. 27915184 10.1016/j.pharep.2016.09.014

[29] Bar A, Olkowicz M, Tyrankiewicz U, Kus E, Jasinski K, Smolenski RT, Skorka T, Chlopicki S. Functional and biochemical endothelial profiling *in vivo* in a murine model of endothelial dysfunction; comparison of effects of 1-methylnicotinamide and angiotensin-converting enzyme inhibitor. Front Pharmacol. 2017;10(8):183. 10.3389/fphar.2017.00183. 10.3389/fphar.2017.00183 PMC5385379 28443021

[30] Ishibashi S, Herz J, Maeda N, Goldstein JL, Brown MS. The two-receptor model of lipoprotein clearance: tests of the hypothesis in “knockout” mice lacking the low density lipoprotein receptor, apolipoprotein E, or both proteins. Proc Natl Acad Sci U S A. 1994;91(10):4431–5. 10.1073/pnas.91.10.4431. 8183926 10.1073/pnas.91.10.4431 PMC43799

[31] Kij A, Bar A, Przyborowski K, Proniewski B, Mateuszuk L, Jasztal A, Kieronska-Rudek A, Marczyk B, Matyjaszczyk-Gwarda K, Tworzydlo A, Enggaard C, Hansen PBL, Jensen B, Walczak M, Chlopicki S. Thrombin inhibition prevents endothelial dysfunction and reverses 20-HETE overproduction without affecting blood pressure in angiotensin II-induced hypertension in mice. Int J Mol Sci. 2021;22(16):8664. 10.3390/ijms22168664. 34445374 10.3390/ijms22168664 PMC8395447

[32] Bar A, Kieronska-Rudek A, Proniewski B, Suraj-Prażmowska J, Czamara K, Marczyk B, Matyjaszczyk-Gwarda K, Jasztal A, Kuś E, Majka Z, Kaczor A, Kurpińska A, Walczak M, Pieterman EJ, Princen HMG, Chlopicki S. In vivo magnetic resonance imaging-based detection of heterogeneous endothelial response in thoracic and abdominal aorta to short-term high-fat diet ascribed to differences in perivascular adipose tissue in mice. J Am Heart Assoc. 2020 3;9(21):e016929. 10.1161/JAHA.120.016929 10.1161/JAHA.120.016929 PMC7763398 33073641

[33] Bar A, Targosz-Korecka M, Suraj J, Proniewski B, Jasztal A, Marczyk B, Sternak M, Przybyło M, Kurpińska A, Walczak M, Kostogrys RB, Szymonski M, Chlopicki S. Degradation of glycocalyx and multiple manifestations of endothelial dysfunction coincide in the early phase of endothelial dysfunction before atherosclerotic plaque development in apolipoprotein E/low-density lipoprotein receptor-deficient mice. J Am Heart Assoc. 2019;8(6):e011171. 10.1161/JAHA.118.011171. 30866689 10.1161/JAHA.118.011171 PMC6475045

[34] Proniewski B, Kij A, Sitek B, Kelley EE, Chlopicki S. Multiorgan development of oxidative and nitrosative stress in LPS-induced endotoxemia in C57Bl/6 mice: DHE-based *in vivo* approach. Oxid Med Cell Longev. 2019;22(2019):7838406. 10.1155/2019/7838406. 10.1155/2019/7838406 PMC6556324 31249650

[35] Sternak M, Bar A, Adamski MG, Mohaissen T, Marczyk B, Kieronska A, Stojak M, Kus K, Tarjus A, Jaisser F, Chlopicki S. The deletion of endothelial sodium channel α (αENaC) impairs endothelium-dependent vasodilation and endothelial barrier integrity in endotoxemia *in vivo*. Front Pharmacol. 2018;10(9):178. 10.3389/fphar.2018.00178. 10.3389/fphar.2018.00178 PMC5902527 29692722

[36] Mohaissen T, Proniewski B, Targosz-Korecka M, Bar A, Kij A, Bulat K, Wajda A, Blat A, Matyjaszczyk-Gwarda K, Grosicki M, Tworzydlo A, Sternak M, Wojnar-Lason K, Rodrigues-Diez R, Kubisiak A, Briones A, Marzec KM, Chlopicki S. Temporal relationship between systemic endothelial dysfunction and alterations in erythrocyte function in a murine model of chronic heart failure. Cardiovasc Res. 2022;118(12):2610-2624. 10.1093/cvr/cvab306. Erratum in: Cardiovasc Res. 2022 Sep 15. 10.1093/cvr/cvab306 PMC9491865 34617995

[37] Fedorowicz A, Buczek E, Mateuszuk Ł, Czarnowska E, Sitek B, Jasztal A, Chmura-Skirlińska A, Dib M, Steven S, Daiber A, Chlopicki S. Comparison of pulmonary and systemic NO- and PGI_2_-dependent endothelial function in diabetic mice. Oxid Med Cell Longev. 2018;4(2018):4036709. 10.1155/2018/4036709. 10.1155/2018/4036709 PMC6008763 29967661

[38] Proniewski B, Bar A, Kieronska-Rudek A, Suraj-Prażmowska J, Buczek E, Czamara K, Majka Z, Czyzynska-Cichon I, Kwiatkowski G, Matyjaszczyk-Gwarda K, Chlopicki S. Systemic administration of insulin receptor antagonist results in endothelial and perivascular adipose tissue dysfunction in mice. Cells. 2021;10(6):1448. 10.3390/cells10061448. 34207844 10.3390/cells10061448 PMC8230211

[39] Panek A, Miszczyk J, Swakoń J. Biological effects and inter-individual variability in peripheral blood lymphocytes of healthy donors exposed to 60 MeV proton radiotherapeutic beam. Int J Radiat Biol. 2018;94(12):1085–94. 10.1080/09553002.2019.1524941. 30273081 10.1080/09553002.2019.1524941

[40] Liu Y, Drozdov I, Shroff R, Beltran LE, Shanahan CM. Prelamin A accelerates vascular calcification via activation of the DNA damage response and senescence-associated secretory phenotype in vascular smooth muscle cells. Circ Res. 2013;112(10):e99-109. 10.1161/CIRCRESAHA.111.300543. 23564641 10.1161/CIRCRESAHA.111.300543

[41] Ragnauth CD, Warren DT, Liu Y, McNair R, Tajsic T, Figg N, Shroff R, Skepper J, Shanahan CM. Prelamin A acts to accelerate smooth muscle cell senescence and is a novel biomarker of human vascular aging. Circulation. 2010;121(20):2200–10. 10.1161/CIRCULATIONAHA.109.902056. 20458013 10.1161/CIRCULATIONAHA.109.902056

[42] Cobb AM, Larrieu D, Warren DT, Liu Y, Srivastava S, Smith AJO, Bowater RP, Jackson SP, Shanahan CM. Prelamin A impairs 53BP1 nuclear entry by mislocalizing NUP153 and disrupting the Ran gradient. Aging Cell. 2016;15(6):1039–50. 10.1111/acel.12506. 27464478 10.1111/acel.12506 PMC5114580

[43] Cobb AM, Murray TV, Warren DT, Liu Y, Shanahan CM. Disruption of PCNA-lamins A/C interactions by prelamin A induces DNA replication fork stalling. Nucleus. 2016;7(5):498–511. 10.1080/19491034.2016.1239685. 27676213 10.1080/19491034.2016.1239685 PMC5120601

[44] Larrieu D, Viré E, Robson S, Breusegem SY, Kouzarides T, Jackson SP. Inhibition of the acetyltransferase NAT10 normalizes progeric and aging cells by rebalancing the Transportin-1 nuclear import pathway. Sci Signal. 2018;11(537):eaar5401. 10.1126/scisignal.aar5401 10.1126/scisignal.aar5401 PMC6331045 29970603

[45] Wiśniewski JR, Zougman A, Nagaraj N, Mann M. Universal sample preparation method for proteome analysis. Nat Methods. 2009;6(5):359–62. 10.1038/nmeth.1322. 19377485 10.1038/nmeth.1322

[46] Meyrelles SS, Peotta VA, Pereira TM, Vasquez EC. Endothelial dysfunction in the apolipoprotein E-deficient mouse: insights into the influence of diet, gender and aging. Lipids Health Dis. 2011;14(10):211. 10.1186/1476-511X-10-211. 10.1186/1476-511X-10-211 PMC3247089 22082357

[47] Bar A, Skórka T, Jasiński K, Sternak M, Bartel Ż, Tyrankiewicz U, Chlopicki S. Retrospectively gated MRI for in vivo assessment of endothelium-dependent vasodilatation and endothelial permeability in murine models of endothelial dysfunction. NMR Biomed. 2016;29(8):1088–97. 10.1002/nbm.3567. 27348596 10.1002/nbm.3567

[48] Yentrapalli R, Azimzadeh O, Barjaktarovic Z, Sarioglu H, Wojcik A, Harms-Ringdahl M, Atkinson MJ, Haghdoost S, Tapio S. Quantitative proteomic analysis reveals induction of premature senescence in human umbilical vein endothelial cells exposed to chronic low-dose rate gamma radiation. Proteomics. 2013;13(7):1096–107. 10.1002/pmic.201200463. 23349028 10.1002/pmic.201200463

[49] Whitmore A, Freeny D, Sojourner SJ, Miles JS, Graham WM, Flores-Rozas H. Evaluation of the role of human DNAJAs in the response to cytotoxic chemotherapeutic agents in a yeast model system. Biomed Res Int. 2020;13(2020):9097638. 10.1155/2020/9097638. 10.1155/2020/9097638 PMC7042521 32149145

[50] Li J, Wang QE, Zhu Q, El-Mahdy MA, Wani G, Praetorius-Ibba M, Wani AA. DNA damage binding protein component DDB1 participates in nucleotide excision repair through DDB2 DNA-binding and cullin 4A ubiquitin ligase activity. Cancer Res. 2006;66(17):8590–7. 10.1158/0008-5472.CAN-06-1115. 16951172 10.1158/0008-5472.CAN-06-1115

[51] Kriger D, Novitskaya K, Vasileva G, Lomert E, Aksenov ND, Barlev NA, Tentler D. Alpha-actnin-4 (ACTN4) selectively affects the DNA double-strand breaks repair in non-small lung carcinoma cells. Biol Direct. 2022;17(1):40. 10.1186/s13062-022-00354-6. 36476259 10.1186/s13062-022-00354-6 PMC9730676

[52] Nakada S, Tai I, Panier S, Al-Hakim A, Iemura S, Juang YC, O’Donnell L, Kumakubo A, Munro M, Sicheri F, Gingras AC, Natsume T, Suda T, Durocher D. Non-canonical inhibition of DNA damage-dependent ubiquitination by OTUB1. Nature. 2010;466(7309):941–6. 10.1038/nature09297. 20725033 10.1038/nature09297

[53] Ehlén Å, Nodin B, Rexhepaj E, Brändstedt J, Uhlén M, Alvarado-Kristensson M, Pontén F, Brennan DJ, Jirström K. RBM3-regulated genes promote DNA integrity and affect clinical outcome in epithelial ovarian cancer. Transl Oncol. 2011;4(4):212–21. 10.1593/tlo.11106. 21804916 10.1593/tlo.11106 PMC3140008

[54] Chetty C, Dontula R, Gujrati M, Dinh DH, Lakka SS. Blockade of SOX4 mediated DNA repair by SPARC enhances radioresponse in medulloblastoma. Cancer Lett. 2012;323(2):188–98. 10.1016/j.canlet.2012.04.014. 22542805 10.1016/j.canlet.2012.04.014 PMC3608856

[55] Song KH, Jung SY, Park JI, Ahn J, Park JK, Um HD, Park IC, Hwang SG, Ha H, Song JY. Inhibition of karyopherin-α2 augments radiation-induced cell death by perturbing BRCA1-mediated DNA repair. Int J Mol Sci. 2019;20(11):2843. 10.3390/ijms20112843. 31212646 10.3390/ijms20112843 PMC6600173

[56] Byrne A, McLaren RP, Mason P, Chai L, Dufault MR, Huang Y, Liang B, Gans JD, Zhang M, Carter K, Gladysheva TB, Teicher BA, Biemann HP, Booker M, Goldberg MA, Klinger KW, Lillie J, Madden SL, Jiang Y. Knockdown of human deubiquitinase PSMD14 induces cell cycle arrest and senescence. Exp Cell Res. 2010;316(2):258–71. 10.1016/j.yexcr.2009.08.018. 19732767 10.1016/j.yexcr.2009.08.018

[57] Guo Y, Zhou A, Zhang Y, Chen Y, Chen Y, Gao Y, Miao X. Serum response factor activates peroxidasin transcription to block senescence of hepatic stellate cells. Life Sci. 2023;328:121824. 10.1016/j.lfs.2023.121824. 37270170 10.1016/j.lfs.2023.121824

[58] Dasgupta J, Kar S, Liu R, Joseph J, Kalyanaraman B, Remington SJ, Chen C, Melendez JA. Reactive oxygen species control senescence-associated matrix metalloproteinase-1 through c-Jun-N-terminal kinase. J Cell Physiol. 2010;225(1):52–62. 10.1002/jcp.22193. 20648623 10.1002/jcp.22193 PMC2913426

[59] Wang T, Zhou LY, Li XM, Liu F, Liang L, Chen XZ, Ju J, Ponnusamy M, Wang K, Liu CY, Yan KW, Wang K. ABRO1 arrests cardiomyocyte proliferation and myocardial repair by suppressing PSPH. Mol Ther. 2023;31(3):847–65. 10.1016/j.ymthe.2023.01.011. 36639869 10.1016/j.ymthe.2023.01.011 PMC10014284

[60] Klement K, Melle C, Murzik U, Diekmann S, Norgauer J, Hemmerich P. Accumulation of annexin A5 at the nuclear envelope is a biomarker of cellular aging. Mech Ageing Dev. 2012;133(7):508–22. 10.1016/j.mad.2012.06.003. 22728018 10.1016/j.mad.2012.06.003

[61] Zhou Y, Chu L, Wang Q, Dai W, Zhang X, Chen J, Li L, Ding P, Zhang L, Gu H, Li L, Lv X, Zhang W, Zhou D, Zhang P, Cai G, Zhao K, Hu W. CD59 is a potential biomarker of esophageal squamous cell carcinoma radioresistance by affecting DNA repair. Cell Death Dis. 2018;9(9):887. 10.1038/s41419-018-0895-0. 30166523 10.1038/s41419-018-0895-0 PMC6117325

[62] Poblocka M, Bassey AL, Smith VM, Falcicchio M, Manso AS, Althubiti M, Sheng X, Kyle A, Barber R, Frigerio M, Macip S. Targeted clearance of senescent cells using an antibody-drug conjugate against a specific membrane marker. Sci Rep. 2021;11(1):20358. 10.1038/s41598-021-99852-2. 34645909 10.1038/s41598-021-99852-2 PMC8514501

[63] Prats H, Martin B, Claverys JP. The hexB mismatch repair gene of Streptococcus pneumoniae: characterisation, cloning and identification of the product. Mol Gen Genet. 1985;200(3):482–9. 10.1007/BF00425735. 2995767 10.1007/BF00425735

[64] Li X, Ren Z, Huang X, Yu T. LACTB, a metabolic therapeutic target in clinical cancer application. Cells. 2022;11(17):2749. 10.3390/cells11172749. 36078157 10.3390/cells11172749 PMC9454609

[65] Piquet S, Le Parc F, Bai SK, Chevallier O, Adam S, Polo SE. The histone chaperone FACT coordinates H2A.X-dependent signaling and repair of DNA damage. Mol Cell. 2018;72(5):888–901.e7. 10.1016/j.molcel.2018.09.010 10.1016/j.molcel.2018.09.010 PMC6292839 30344095

[66] Uchihara Y, Permata TBM, Sato H, Kawabata-Iwakawa R, Katada S, Gu W, Kakoti S, Yamauchi M, Kato R, Gondhowiardjo S, Hosen N, Yasuhara T, Shibata A. DNA damage promotes HLA class I presentation by stimulating a pioneer round of translation-associated antigen production. Mol Cell. 2022;82(14):2557-2570.e7. 10.1016/j.molcel.2022.04.030. 35594857 10.1016/j.molcel.2022.04.030

[67] Zhang W, Lin L, Xia L, Cai W, Dai W, Zou C, Yin L, Tang D, Xu Y, Dai Y. Multi-omics analyses of human colorectal cancer revealed three mitochondrial genes potentially associated with poor outcomes of patients. J Transl Med. 2021;19(1):273. 10.1186/s12967-021-02939-7. 34174878 10.1186/s12967-021-02939-7 PMC8236205

[68] Min S, Kwon SM, Hong J, Lee YK, Park TJ, Lim SB, Yoon G. Mitoribosomal deregulation drives senescence via TPP1-mediated telomere deprotection. Cells. 2022;11(13):2079. 10.3390/cells11132079. 35805162 10.3390/cells11132079 PMC9265344

[69] Cabello-Lobato MJ, Wang S, Schmidt CK. SAMHD1 sheds moonlight on DNA double-strand break repair. Trends Genet. 2017;33(12):895–7. 10.1016/j.tig.2017.09.007. 28969870 10.1016/j.tig.2017.09.007

[70] Rovira M, Sereda R, Pladevall-Morera D, Ramponi V, Marin I, Maus M, Madrigal-Matute J, Díaz A, García F, Muñoz J, Cuervo AM, Serrano M. The lysosomal proteome of senescent cells contributes to the senescence secretome. Aging Cell. 2022;21(10):e13707. 10.1111/acel.13707. 36087066 10.1111/acel.13707 PMC9577959

[71] Rizvi F, Preston CC, Emelyanova L, Yousufuddin M, Viqar M, Dakwar O, Ross GR, Faustino RS, Holmuhamedov EL, Jahangir A. Effects of aging on cardiac oxidative stress and transcriptional changes in pathways of reactive oxygen species generation and clearance. J Am Heart Assoc. 2021;10(16):e019948. 10.1161/JAHA.120.019948. 34369184 10.1161/JAHA.120.019948 PMC8475058

[72] Liu B, Meng Q, Gao X, Sun H, Xu Z, Wang Y, Zhou H. Lipid and glucose metabolism in senescence. Front Nutr. 2023;23(10):1157352. 10.3389/fnut.2023.1157352. 10.3389/fnut.2023.1157352 PMC10481967 37680899

[73] Saul D, Kosinsky RL, Atkinson EJ, Doolittle ML, Zhang X, LeBrasseur NK, Pignolo RJ, Robbins PD, Niedernhofer LJ, Ikeno Y, Jurk D, Passos JF, Hickson LJ, Xue A, Monroe DG, Tchkonia T, Kirkland JL, Farr JN, Khosla S. A new gene set identifies senescent cells and predicts senescence-associated pathways across tissues. Nat Commun. 2022;13(1):4827. 10.1038/s41467-022-32552-1. 35974106 10.1038/s41467-022-32552-1 PMC9381717

[74] Jin Y, Zhao L, Wang S, Zhang X, Quan J, Lin Z, Piao J. RSL1D1 knockdown induces ferroptosis and mediates ferrous iron accumulation in senescent cells by inhibiting FTH1 mRNA stability. Carcinogenesis. 2023;44(2):129–42. 10.1093/carcin/bgad012. 36913375 10.1093/carcin/bgad012

[75] Choy B, LaLonde A, Que J, Wu T, Zhou Z. MCM4 and MCM7, potential novel proliferation markers, significantly correlated with Ki-67, Bmi1, and cyclin E expression in esophageal adenocarcinoma, squamous cell carcinoma, and precancerous lesions. Hum Pathol. 2016;57:126–35. 10.1016/j.humpath.2016.07.013. 27476776 10.1016/j.humpath.2016.07.013 PMC5250507

[76] Matsudaira T, Nakano S, Konishi Y, Kawamoto S, Uemura K, Kondo T, Sakurai K, Ozawa T, Hikida T, Komine O, Yamanaka K, Fujita Y, Yamashita T, Matsumoto T, Hara E. Cellular senescence in white matter microglia is induced during ageing in mice and exacerbates the neuroinflammatory phenotype. Commun Biol. 2023;6(1):665. 10.1038/s42003-023-05027-2. 37353538 10.1038/s42003-023-05027-2 PMC10290132

[77] Young JJ, Patel A, Rai P. Suppression of thioredoxin-1 induces premature senescence in normal human fibroblasts. Biochem Biophys Res Commun. 2010;392(3):363–8. 10.1016/j.bbrc.2010.01.026. 20074557 10.1016/j.bbrc.2010.01.026

[78] Peng H, Guo Q, Xiao Y, Su T, Jiang TJ, Guo LJ, Wang M. *ASPH* regulates osteogenic differentiation and cellular senescence of BMSCs. Front Cell Dev Biol. 2020;3(8):872. 10.3389/fcell.2020.00872. 10.3389/fcell.2020.00872 PMC7494742 33015050

[79] Jiao S, Meng F, Zhang J, Yang X, Zheng X, Wang L. STAT1 mediates cellular senescence induced by angiotensin II and H_2_O_2_ in human glomerular mesangial cells. Mol Cell Biochem. 2012;365(1–2):9–17. 10.1007/s11010-011-1197-3. 22193460 10.1007/s11010-011-1197-3

[80] Mittermeier C, Konopa A, Muehlich S. Molecular mechanisms to target cellular senescence in hepatocellular carcinoma. Cells. 2020;9(12):2540. 10.3390/cells9122540. 33255630 10.3390/cells9122540 PMC7761055

[81] You GR, Chang JT, Li YL, Huang CW, Tsai YL, Fan KH, Kang CJ, Huang SF, Chang PH, Cheng AJ. MYH9 facilitates cell invasion and radioresistance in head and neck cancer via modulation of cellular ROS levels by activating the MAPK-Nrf2-GCLC pathway. Cells. 2022;11(18):2855. 10.3390/cells11182855. 36139430 10.3390/cells11182855 PMC9497050

[82] Buljan M, Ciuffa R, van Drogen A, Vichalkovski A, Mehnert M, Rosenberger G, Lee S, Varjosalo M, Pernas LE, Spegg V, Snijder B, Aebersold R, Gstaiger M. Kinase interaction network expands functional and disease roles of human kinases. Mol Cell. 2020;79(3):504-520.e9. 10.1016/j.molcel.2020.07.001. 32707033 10.1016/j.molcel.2020.07.001 PMC7427327

[83] Lou Z, Wei J, Riethman H, Baur JA, Voglauer R, Shay JW, Wright WE. Telomere length regulates ISG15 expression in human cells. Aging (Albany NY). 2009;1(7):608–21. 10.18632/aging.100066. 20157543 10.18632/aging.100066 PMC2806043

[84] Kim HY, Hong S. Multi-faceted roles of DNAJB protein in cancer metastasis and clinical implications. Int J Mol Sci. 2022;23(23):14970. 10.3390/ijms232314970. 36499297 10.3390/ijms232314970 PMC9737691

[85] Ghodke I, Remisova M, Furst A, Kilic S, Reina-San-Martin B, Poetsch AR, Altmeyer M, Soutoglou E. AHNAK controls 53BP1-mediated p53 response by restraining 53BP1 oligomerization and phase separation. Mol Cell. 2021;81(12):2596-2610.e7. 10.1016/j.molcel.2021.04.010. 33961796 10.1016/j.molcel.2021.04.010 PMC8221568

[86] Song D, Shang J, Long Y, Zhong M, Li L, Chen J, Xiang Y, Tan H, Zhu H, Hong X, Hou FF, Fu H, Liu Y. Insulin-like growth factor 2 mRNA-binding protein 3 promotes kidney injury by regulating β-catenin signaling. JCI Insight. 2023;8(2):e162060. 10.1172/jci.insight.162060. 36520532 10.1172/jci.insight.162060 PMC9977311

[87] Lv J, Hu Y, Li L, He Y, Wang J, Guo N, Fang Y, Chen Q, Cai C, Tong J, Tang L, Wang Z. Targeting FABP4 in elderly mice rejuvenates liver metabolism and ameliorates aging-associated metabolic disorders. Metabolism. 2023;142:155528. 10.1016/j.metabol.2023.155528. 36842611 10.1016/j.metabol.2023.155528

[88] Wang J, Yang B, Wang D, Han R, Bi Z, Lin L. GARS is implicated in poor survival and immune infiltration of hepatocellular carcinoma. Cell Signal. 2022;94:110302. 10.1016/j.cellsig.2022.110302. 35271987 10.1016/j.cellsig.2022.110302

[89] Wardlaw CP, Petrini JHJ. ISG15 conjugation to proteins on nascent DNA mitigates DNA replication stress. Nat Commun. 2022;13(1):5971. 10.1038/s41467-022-33535-y. 36216822 10.1038/s41467-022-33535-y PMC9550767

[90] Kang D, Lee J, Jung J, Carlson BA, Chang MJ, Chang CB, Kang SB, Lee BC, Gladyshev VN, Hatfield DL, Lee BJ, Kim JH. Selenophosphate synthetase 1 deficiency exacerbates osteoarthritis by dysregulating redox homeostasis. Nat Commun. 2022;13(1):779. 10.1038/s41467-022-28385-7. 35140209 10.1038/s41467-022-28385-7 PMC8828855

[91] Wang R, Yang Y, Zhang Z, Zhao N, Wiemer EAC, Ben J, Ma J, Yuan L. Major vault protein (MVP) suppresses aging- and estrogen deficiency-related bone loss through Fas-mediated apoptosis in osteoclasts. Cell Death Dis. 2023;14(9):604. 10.1038/s41419-023-05928-4. 37704623 10.1038/s41419-023-05928-4 PMC10500014

[92] Cobb AM, Yusoff S, Hayward R, Ahmad S, Sun M, Verhulst A, D′Haese PC, Shanahan CM. Runx2 (runt-related transcription factor 2) links the DNA damage response to osteogenic reprogramming and apoptosis of vascular smooth muscle cells. Arterioscler Thromb Vasc Biol. 2021;41(4):1339–1357. 10.1161/ATVBAHA.120.315206. Erratum in: Arterioscler Thromb Vasc Biol. 2021 Oct;41(10):e497. 10.1161/ATVBAHA.120.315206 33356386

[93] Shea MK, Booth SL, Weiner DE, Brinkley TE, Kanaya AM, Murphy RA, Simonsick EM, Wassel CL, Vermeer C, Kritchevsky SB; Health ABC Study. Circulating vitamin K is inversely associated with incident cardiovascular disease risk among those treated for hypertension in the health, aging, and body composition study (Health ABC). J Nutr. 2017;147(5):888–895. 10.3945/jn.117.249375. 10.3945/jn.117.249375 PMC5404216 28356433

[94] Dupuy M, Radavelli-Bagatini S, Zhong L, Dalla Via J, Zhu K, Blekkenhorst LC, Bondonno NP, Linneberg A, Bellinge JW, Schultz C, Courtney W, Prince RL, Hodgson JM, Lewis JR, Sim M. Vitamin K1 intake is associated with lower risk for all-cause and cardiovascular disease mortality in community-dwelling older Australian women. Nutr Metab Cardiovasc Dis. 2023:S0939–4753(23)00501-X. 10.1016/j.numecd.2023.12.007 10.1016/j.numecd.2023.12.007 38342722

[95] Villines TC, Hatzigeorgiou C, Feuerstein IM, O’Malley PG, Taylor AJ. Vitamin K1 intake and coronary calcification. Coron Artery Dis. 2005;16(3):199–203. 10.1097/00019501-200505000-00010. 15818090 10.1097/00019501-200505000-00010

[96] Bellinge JW, Dalgaard F, Murray K, Connolly E, Blekkenhorst LC, Bondonno CP, Lewis JR, Sim M, Croft KD, Gislason G, Torp-Pedersen C, Tjønneland A, Overvad K, Hodgson JM, Schultz C, Bondonno NP. Vitamin K Intake and atherosclerotic cardiovascular disease in the Danish Diet Cancer and Health study. J Am Heart Assoc. 2021;10(16):e020551. 10.1161/JAHA.120.020551. 34369182 10.1161/JAHA.120.020551 PMC8475061

[97] Wojewoda M, Tyrankiewicz U, Gwozdz P, Skorka T, Jablonska M, Orzylowska A, Jasinski K, Jasztal A, Przyborowski K, Kostogrys RB, Zoladz JA, Chlopicki S. Exercise capacity and cardiac hemodynamic response in female ApoE/LDLR(-/-) mice: a paradox of preserved V’O2max and exercise capacity despite coronary atherosclerosis. Sci Rep. 2016;25(6):24714. 10.1038/srep24714. 10.1038/srep24714 PMC4842974 27108697

[98] Tyrankiewicz U, Skorka T, Orzylowska A, Jablonska M, Jasinski K, Jasztal A, Bar A, Kostogrys R, Chlopicki S. Comprehensive MRI for the detection of subtle alterations in diastolic cardiac function in apoE/LDLR(-/-) mice with advanced atherosclerosis. NMR Biomed. 2016;29(6):833–40. 10.1002/nbm.3524. 27146203 10.1002/nbm.3524

[99] Olkowicz M, Rosales-Solano H, Ramadan K, Wang A, Cypel M, Pawliszyn J. The metabolic fate of oxaliplatin in the biological milieu investigated during *in vivo* lung perfusion using a unique miniaturized sampling approach based on solid-phase microextraction coupled with liquid chromatography-mass spectrometry. Front Cell Dev Biol. 2022;25(10):928152. 10.3389/fcell.2022.928152. 10.3389/fcell.2022.928152 PMC9453651 36092704

[100] Ebina K, Shi K, Hirao M, Kaneshiro S, Morimoto T, Koizumi K, Yoshikawa H, Hashimoto J. Vitamin K2 administration is associated with decreased disease activity in patients with rheumatoid arthritis. Mod Rheumatol. 2013;23(5):1001–7. 10.1007/s10165-012-0789-4. 23124653 10.1007/s10165-012-0789-4

[101] Okamoto H, Shidara K, Hoshi D, Kamatani N. Anti-arthritis effects of vitamin K(2) (menaquinone-4)–a new potential therapeutic strategy for rheumatoid arthritis. FEBS J. 2007;274(17):4588–94. 10.1111/j.1742-4658.2007.05987.x. 17681015 10.1111/j.1742-4658.2007.05987.x

[102] Mehdizadeh M, Aguilar M, Thorin E, Ferbeyre G, Nattel S. The role of cellular senescence in cardiac disease: basic biology and clinical relevance. Nat Rev Cardiol. 2022;19(4):250–64. 10.1038/s41569-021-00624-2. 34667279 10.1038/s41569-021-00624-2

[103] Jia G, Aroor AR, Jia C, Sowers JR. Endothelial cell senescence in aging-related vascular dysfunction. Biochim Biophys Acta Mol Basis Dis. 2019;1865(7):1802–9. 10.1016/j.bbadis.2018.08.008. 31109450 10.1016/j.bbadis.2018.08.008

[104] Berkowicz P, Totoń-Żurańska J, Kwiatkowski G, Jasztal A, Csípő T, Kus K, Tyrankiewicz U, Orzyłowska A, Wołkow P, Tóth A, Chlopicki S. Accelerated ageing and coronary microvascular dysfunction in chronic heart failure in Tgαq*44 mice. Geroscience. 2023;45(3):1619–48. 10.1007/s11357-022-00716-y. 36692592 10.1007/s11357-022-00716-y PMC10400753

[105] Chen MS, Lee RT, Garbern JC. Senescence mechanisms and targets in the heart. Cardiovasc Res. 2022;118(5):1173–87. 10.1093/cvr/cvab161. 33963378 10.1093/cvr/cvab161 PMC8953446

[106] Wang J, Uryga AK, Reinhold J, Figg N, Baker L, Finigan A, Gray K, Kumar S, Clarke M, Bennett M. Vascular smooth muscle cell senescence promotes atherosclerosis and features of plaque vulnerability. Circulation. 2015;132(20):1909–19. 10.1161/CIRCULATIONAHA.115.016457. 26416809 10.1161/CIRCULATIONAHA.115.016457

[107] Cunha PG, Boutouyrie P, Nilsson PM, Laurent S. Early vascular ageing (EVA): definitions and clinical applicability. Curr Hypertens Rev. 2017;13(1):8–15. 10.2174/1573402113666170413094319. 28412914 10.2174/1573402113666170413094319

[108] Bancaro N, Calì B, Troiani M, Elia AR, Arzola RA, Attanasio G, Lai P, Crespo M, Gurel B, Pereira R, Guo C, Mosole S, Brina D, D’Ambrosio M, Pasquini E, Spataro C, Zagato E, Rinaldi A, Pedotti M, Di Lascio S, Meani F, Montopoli M, Ferrari M, Gallina A, Varani L, Pereira Mestre R, Bolis M, Gillessen Sommer S, de Bono J, Calcinotto A, Alimonti A. Apolipoprotein E induces pathogenic senescent-like myeloid cells in prostate cancer. Cancer Cell. 2023;41(3):602-619.e11. 10.1016/j.ccell.2023.02.004. 36868226 10.1016/j.ccell.2023.02.004

[109] Komaravolu RK, Waltmann MD, Konaniah E, Jaeschke A, Hui DY. ApoER2 (apolipoprotein E receptor-2) deficiency accelerates smooth muscle cell senescence via cytokinesis impairment and promotes fibrotic neointima after vascular injury. Arterioscler Thromb Vasc Biol. 2019;39(10):2132–44. 10.1161/ATVBAHA.119.313194. 31412739 10.1161/ATVBAHA.119.313194 PMC6761011

[110] Serino A, Zhao Y, Hwang J, Cullen A, Deeb C, Akhavan N, Arjmandi B, Salazar G. Gender differences in the effect of blackberry supplementation in vascular senescence and atherosclerosis in ApoE^−/−^ mice. J Nutr Biochem. 2020;80:108375. 10.1016/j.jnutbio.2020.108375. 32248057 10.1016/j.jnutbio.2020.108375

[111] d'Adda di Fagagna F. Living on a break: Cellular senescence as a DNA-damage response. Nat Rev Cancer. 2008;8(7):512–22. 10.1038/nrc2440. 10.1038/nrc2440 18574463

[112] Salmon TB, Evert BA, Song B, Doetsch PW. Biological consequences of oxidative stress-induced DNA damage in Saccharomyces cerevisiae. Nucleic Acids Res. 2004;32(12):3712–23. 10.1093/nar/gkh696. 15254273 10.1093/nar/gkh696 PMC484183

[113] Bloom SI, Tucker JR, Lim J, Thomas TG, Stoddard GJ, Lesniewski LA, Donato AJ. Aging results in DNA damage and telomere dysfunction that is greater in endothelial versus vascular smooth muscle cells and is exacerbated in atheroprone regions. Geroscience. 2022;44(6):2741–55. 10.1007/s11357-022-00681-6. 36350415 10.1007/s11357-022-00681-6 PMC9768045

[114] Ungvari Z, Tarantini S, Nyúl-Tóth Á, Kiss T, Yabluchanskiy A, Csipo T, Balasubramanian P, Lipecz A, Benyo Z, Csiszar A. Nrf2 dysfunction and impaired cellular resilience to oxidative stressors in the aged vasculature: From increased cellular senescence to the pathogenesis of age-related vascular diseases. Geroscience. 2019;41(6):727–38. 10.1007/s11357-019-00107-w. 31655958 10.1007/s11357-019-00107-w PMC6925097

[115] Kaźmierczak-Barańska J, Karwowski BT. Vitamin K contribution to DNA damage-advantage or disadvantage? A human health response. Nutrients. 2022;14(20):4219. 10.3390/nu14204219. 36296903 10.3390/nu14204219 PMC9611527

[116] Vervoort LM, Ronden JE, Thijssen HH. The potent antioxidant activity of the vitamin K cycle in microsomal lipid peroxidation. Biochem Pharmacol. 1997;54(8):871–6. 10.1016/s0006-2952(97)00254-2. 9354587 10.1016/s0006-2952(97)00254-2

[117] Ngo EO, Sun TP, Chang JY, Wang CC, Chi KH, Cheng AL, Nutter LM. Menadione-induced DNA damage in a human tumor cell line. Biochem Pharmacol. 1991;42(10):1961–8. 10.1016/0006-2952(91)90596-w. 1741774 10.1016/0006-2952(91)90596-w

[118] Li J, Lin JC, Wang H, Peterson JW, Furie BC, Furie B, Booth SL, Volpe JJ, Rosenberg PA. Novel role of vitamin K in preventing oxidative injury to developing oligodendrocytes and neurons. J Neurosci. 2003;23(13):5816–26. 10.1523/JNEUROSCI.23-13-05816.2003. 12843286 10.1523/JNEUROSCI.23-13-05816.2003 PMC6741273

[119] Popa DS, Bigman G, Rusu ME. The role of vitamin K in humans: implication in aging and age-associated diseases. Antioxidants (Basel). 2021;10(4):566. 10.3390/antiox10040566. 33917442 10.3390/antiox10040566 PMC8067486

[120] Mercurio V, Lobasso A, Barbieri L, Parrella P, Ciervo D, Liccardo B, Bonaduce D, Tocchetti CG, De Paulis A, Rossi FW. Inflammatory, serological and vascular determinants of cardiovascular disease in systemic lupus erythematosus patients. Int J Mol Sci. 2019;20(9):2154. 10.3390/ijms20092154. 31052336 10.3390/ijms20092154 PMC6540240

[121] Wolf D, Ley K. Immunity and inflammation in atherosclerosis. Circ Res. 2019;124(2):315–27. 10.1161/CIRCRESAHA.118.313591. 30653442 10.1161/CIRCRESAHA.118.313591 PMC6342482

[122] Golia E, Limongelli G, Natale F, Fimiani F, Maddaloni V, Pariggiano I, Bianchi R, Crisci M, D’Acierno L, Giordano R, Di Palma G, Conte M, Golino P, Russo MG, Calabrò R, Calabrò P. Inflammation and cardiovascular disease: from pathogenesis to therapeutic target. Curr Atheroscler Rep. 2014;16(9):435. 10.1007/s11883-014-0435-z. 25037581 10.1007/s11883-014-0435-z

[123] Jiang Y, Yabluchanskiy A, Deng J, Amil FA, Po SS, Dasari TW. The role of age-associated autonomic dysfunction in inflammation and endothelial dysfunction. Geroscience. 2022;44(6):2655–70. 10.1007/s11357-022-00616-1. 35773441 10.1007/s11357-022-00616-1 PMC9768093

[124] Gogulamudi VR, Durrant JR, Adeyemo AO, Ho HM, Walker AE, Lesniewski LA. Advancing age increases the size and severity of spontaneous atheromas in mouse models of atherosclerosis. Geroscience. 2023;45(3):1913–31. 10.1007/s11357-023-00776-8. 37086367 10.1007/s11357-023-00776-8 PMC10400524

[125] Bobryshev YV, Ivanova EA, Chistiakov DA, Nikiforov NG, Orekhov AN. Macrophages and their role in atherosclerosis: pathophysiology and transcriptome analysis. Biomed Res Int. 2016;2016:9582430. 10.1155/2016/9582430. 27493969 10.1155/2016/9582430 PMC4967433

[126] Hwang SJ, Ballantyne CM, Sharrett AR, Smith LC, Davis CE, Gotto AM Jr, Boerwinkle E. Circulating adhesion molecules VCAM-1, ICAM-1, and E-selectin in carotid atherosclerosis and incident coronary heart disease cases: the Atherosclerosis Risk In Communities (ARIC) study. Circulation. 1997;96(12):4219–25. 10.1161/01.cir.96.12.4219. 9416885 10.1161/01.cir.96.12.4219

[127] Galkina E, Ley K. Vascular adhesion molecules in atherosclerosis. Arterioscler Thromb Vasc Biol. 2007;27(11):2292–301. 10.1161/ATVBAHA.107.149179. 17673705 10.1161/ATVBAHA.107.149179

[128] Wang T, Tian J, Jin Y. VCAM1 expression in the myocardium is associated with the risk of heart failure and immune cell infiltration in myocardium. Sci Rep. 2021;11(1):19488. 10.1038/s41598-021-98998-3. 34593936 10.1038/s41598-021-98998-3 PMC8484263

[129] Huang J, Xu X, Zhou Y, Xin Z, Cao Q, He R, Hou T, Ding Y, Lu J, Wang T, Zhao Z, Xu Y, Wang W, Ning G, Xu M, Wang L, Bi Y, Li M. Age-specific difference in the temporal relationships between updated cardiovascular health construct and arterial stiffness in middle-aged and older adults. Geroscience. 2023. 10.1007/s11357-023-00965-5. 10.1007/s11357-023-00965-5 PMC10828153 37814197

[130] Coll-Bonfill N, Mahajan U, Shashkova EV, Lin CJ, Mecham RP, Gonzalo S. Progerin induces a phenotypic switch in vascular smooth muscle cells and triggers replication stress and an aging-associated secretory signature. Geroscience. 2023;45(2):965–82. 10.1007/s11357-022-00694-1. 36482259 10.1007/s11357-022-00694-1 PMC9886737

[131] Reddi K, Henderson B, Meghji S, Wilson M, Poole S, Hopper C, Harris M, Hodges SJ. Interleukin 6 production by lipopolysaccharide-stimulated human fibroblasts is potently inhibited by naphthoquinone (vitamin K) compounds. Cytokine. 1995;7(3):287–90. 10.1006/cyto.1995.0034. 7640347 10.1006/cyto.1995.0034

[132] Koshihara Y, Hoshi K, Shiraki M. Vitamin K2 (menatetrenone) inhibits prostaglandin synthesis in cultured human osteoblast-like periosteal cells by inhibiting prostaglandin H synthase activity. Biochem Pharmacol. 1993;46(8):1355–62. 10.1016/0006-2952(93)90099-i. 8240383 10.1016/0006-2952(93)90099-i

[133] Schurgers LJ, Vermeer C. Determination of phylloquinone and menaquinones in food. Effect of food matrix on circulating vitamin K concentrations. Haemostasis. 2000;30(6):298–307. 10.1159/000054147 10.1159/000054147 11356998

[134] Thijssen HH, Drittij-Reijnders MJ. Vitamin K status in human tissues: tissue-specific accumulation of phylloquinone and menaquinone-4. Br J Nutr. 1996;75(1):121–7. 10.1079/bjn19960115. 8785182 10.1079/bjn19960115

[135] Schurgers LJ, Vermeer C. Differential lipoprotein transport pathways of K-vitamins in healthy subjects. Biochim Biophys Acta. 2002;1570(1):27–32. 10.1016/s0304-4165(02)00147-2. 11960685 10.1016/s0304-4165(02)00147-2

[136] Siew A, Le H, Thiovolet M, Gellert P, Schatzlein A, Uchegbu I. Enhanced oral absorption of hydrophobic and hydrophilic drugs using quaternary ammonium palmitoyl glycol chitosan nanoparticles. Mol Pharm. 2012;9(1):14–28. 10.1021/mp200469a. 22047066 10.1021/mp200469a

