# Supplementary material for: Phylloquinone improves endothelial function, inhibits cellular senescence, and vascular inflammation
Source: GeroScience. 2024 Jul 9;46(5):4909–35. doi: 10.1007/s11357-024-01225-w (PMC11336140; doi:10.1007/s11357-024-01225-w)
Supplement: Supplementary file 1 — Supplementary file1 (DOCX 23 KB) [file 11357_2024_1225_MOESM1_ESM.docx]

**SUPPLEMENTARY MATERIALS GEROSCIENCE**

**Vascular protective effects of phylloquinone and menaquinone: protection against endothelial dysfunction, inhibition of cellular senescence, and inhibition of vascular inflammation**

Anna Kieronska-Rudek^1,2,3^, Agnieszka Kij^1^, Anna Bar^1^, Anna Kurpinska^1^, Tasnim Mohaissen^1^, Marek Grosicki^1^, Marta Stojak^1^, Magdalena Sternak^1^, Elżbieta Buczek^1^, Bartosz Proniewski^1^, Kamil Kuś^1^, Joanna Suraj-Prazmowska^1^, Agnieszka Panek^4^, Monika Pietrowska^5^, Szczepan Zapotoczny^6^, Catherine M. Shanahan^7^, Csaba Szabo^3^, Stefan Chlopicki^1,2*^

^1^ Jagiellonian University, Jagiellonian Centre for Experimental Therapeutics (JCET), Krakow, Poland,
^2^ Jagiellonian University Medical College, Chair of Pharmacology, Faculty of Medicine, Krakow, Poland,

^3^ University of Fribourg, Chair of Pharmacology, Faculty of Science and Medicine, Fribourg, Switzerland.

^4^ Institute of Nuclear Physics Polish Academy of Sciences, Krakow, Poland

^5^ Centre for Translational Research and Molecular Biology of Cancer, Maria Sklodowska-Curie National Research Institute of Oncology, Gliwice, Poland

^7^ Jagiellonian University, Department of Physical Chemistry and Electrochemistry, Faculty of Chemistry, Krakow, Poland

^7^ King's College London, School of Cardiovascular and Metabolic Medicine and Sciences, James Black Centre, London, United Kingdom

*Corresponding author:

Stefan Chlopicki

Email: stefan.chlopicki@jcet.eu

***Supplementary Methods:***

**1. Proteomic analysis of changes in protein profile associated with cell senescence**

Microcon Ultracel 30 kDa (Merck, Darmstadt) filters were prepared by double spinning using 14,000 x g, 15 min. 20°C with 8M urea in 100 mM Tris-HCl (pH 8) (UA). Aliquots of 75 µg of protein were mixed with UA and transferred into individual filters. After centrifugation, an additional 200 µl of UA was added to the filters and centrifuged again. Disulphide bonds were reduced with 50 µl of 50 mM DL-dithiotreitol (BioShop, Burlington, Ontario, Canada) in UA (56°C, 20 min) and alkylated with 50 µl of 50 mM iodoacetamide (Sigma Aldrich, Saint Louis, Missouri, USA) in UA (protected from light, 20°C, 20 min), each time centrifuged as mentioned above. Double washing step with 100 µl of UA and double washing step with 100 µl of 40 mM ammonium bicarbonate (ABC) (Sigma Aldrich, Saint Louis, Missouri, USA) was applied. Proteins were first digested with Lys-C (18h, 37°C, 1:100; New England BioLabs, Ipswich, Massachusetts), digested peptides and eluate after rinsing with 40 mM ABC were collected (10,000 x g, 10 min., RT). Digestion was quenched with 0.5% TFA (Sigma Aldrich, Germany). The remaining proteins on the filter were subsequently digested with trypsin (3 h, 37°C, 1:100; Promega, Madison, WI). Eluates were analyzed by MS separately. Peptides were purified using Pierce Peptide Desalting Spin Columns according to the manufacturer's instructions (Thermo Scientific, USA). The peptide concentration was assessed using a modified BCA assay (Thermo Scientific, Germany).

Fractions were analyzed using an LC-MS system composed of nano-HPLC – Ultimate 3000 (Dionex) directly coupled to a high-resolution Q Exactive Plus Hybrid Quadrupole Orbitrap mass spectrometer (Thermo Scientific, USA). Chromatography was carried out at a flow rate 300 nL/min using 0.1%FA in LC-MS grade water (as mobile phase A) and 80% acetonitrile with 0.1% FA in LC-MS grade water (as mobile phase B) at 30^0^C. The peptides were separated (for200min) in the 180 min preformed gradient on a reverse-phase Acclaim PepMap RSLC nanoViper C18 analytical column (0.075 mm diameter, 500 mm length, 2 µm particle size, 100 Å pore size). For additional desalting purposes, the samples were loaded onto a PepMap Neo Trap Cartridge (300 µm diameter, 5 mm length, 5 µm particle size, 100 Å pore size) for 3 min using 0.1% FA in LC-MS grade water as a loading buffer. After desalting, the trap column was switched with the analytical column and the peptides were eluted using the binary gradients of 3–8% of mobile phase B for 7min, 8–35% of mobile phase B for 140 min and 35–60% of mobile phase B for a further 20 min. Finally, rinsing the column with 80% of mobile phase B for 20 min and equilibration in 3% of mobile phase B for another 20 min were performed. Data were acquired in positive mode with a data-dependent method using the following parameters. MS1 resolution was set at 70,000 at 200 m/z with a normalized AGC target 3e6, auto maximum inject time of 50 ms and a scan range of 350 to 1500 m/z. For MS2, a resolution was set at 17,500 at 200 m/z with a standard normalized AGC target. Auto maximum inject time and top 12 precursors within an isolation window of 1.4 m/z were considered for MS/MS analysis. Dynamic exclusion was set at 30 s and the precursor intensity threshold at 2.5e5. Precursors were fragmented in HCD mode with a normalized collision energy of 35 %. Multivariate analyses were carried out by untargeted principal component analysis (PCA). All statistical analyses (ANOVA with post hoc Tukey’s test, False Discover Rate (FDR): q≤0.05), visualization of the results and functional analysis were performed using Perseus version 2.0.7.0 (Max Planck Institute of Biochemistry, Martinsried, Germany) ^39^ STRING,^40-41^ ShinyGO,^42^ Reactome,^43^ InteractiVenn^44-48^ together with manual search for pathway and functional allocation of the identified differentially regulated proteins.

1. **Analysis of the influence of PK and MK on the proliferation of senescent cells**

A real-time cell-based wound healing assay combined with quantitative analysis was performed using the 96W1E+ ECIS array (Applied BioPhysics, Troy, NY) according to a previous protocol.^49^ Prior to seeding the cells, the 96W1E+ plate was pretreated for 10 minutes with L-cysteine (10 mM), (Sigma-Aldrich, Steinheim, Germany) at room temperature and washed twice with ultrapure water. After washing, 200 μl of DMEM medium was added to each well to check the basic values of resistance (Ω), capacitance (μF) and impedance (Ω). Then both PAEC and HAEC cells were seeded at a density of 3.5 x 104 per well in a final volume of 300 μl. Resistance, capacitance, and impedance values were recorded at frequencies from 250 Hz to 64 kHz (250, 500, 1000, 2000, 4000, 8000, 16,000, 32,000, and 64,000 Hz) using the time-multi-frequency mode. Cell-free wells served as negative controls to ensure baseline impedance changes for all experiments. When the impedance signal reached a stationary value (2–3 days after cell seeding), the "wound" was burned off by applying 3000 µA, 4 kHz AC for 30 seconds, killing the cells on the electrode surface. Dead cells were washed away with DPBS, and wound healing was assessed by continuous resistance measurements for 48 hours in the presence of 5 or 10 µM PK or MK. The medium was changed every 24 h to ensure a constant concentration of vitamin K. The experiment was performed in a humidified 5% CO_2_ incubator at 37°C and repeated three times in 3-4 technical replicates. The area under the curve (AUC) was quantified and the results were normalized to the untreated control.

1. **Analysis of changes in gene expression associated with cell senescence by RT-PCR.**

Changes in gene expression associated with senescence were determined using quantitative real-time polymerase chain reaction (RT-PCR) with reverse transcription as described in detail in **Supplementary Methods**. The total RNA pool was isolated from the cells using RNA-STAT-60 (Amsbio, Cambridge, MA, USA), according to the manufacturer's protocol. Reverse transcription was then performed using the GoScript™ Reverse Transcriptase kit (Promega, Madison, WI, USA). Quantitative RT-PCR was performed on an Applied Biosystems StepOne thermal cycler (Applied Biosystems, Foster City, CA, USA) with an SYBR™ Green PCR Master Mix fluorescent probe (Applied Bioscience, Carlsbad, CA, USA) for quantitative product monitoring reaction. The reaction was carried out in a volume of 20 μl of the reaction mixture. The following cycle settings were used: 95°C for 10 min followed by 40 cycles of 95°C for 5 sec and 60°C for 1 min. The expression of genes related to cell senescence (p16^(INK4a)^/CDKN2A, p21/CDKN1A, and IL-6) was assessed using specific primers listed below. *Results were normalized to the expression of the 18S ribosomal RNA subunit gene; reference gene (Housekeeping gene) selected based on stable expression in response to the treatment with studied compounds* **(Supplementary Table 1).**

**Supplementary Table 1. Proteomic data obtained from LC-MS analysis, that has been visualized on graphs in Fig.4 and Fig. 5.**

| ***PCR Primers*** | |
| --- | --- |
| 18S | QT00199367 Hs_RRN18S_1_SG QuantiTect Primer Assay |
| p21/ CDKN1A | QT00062090 Hs_CDKN1A_1_SG QuantiTect Primer Assay |
| p16^(INK4a)^/ CDKN2A | QT00998459 Hs_CDKN2A_vb.1_SG QuantiTect Primer Assay |
| IL-6 | QT00083720 Hs_IL6_1_SG QuantiTect Primer Assay |

1. **Immunocytochemical analysis**

Cells were washed with DPBS (Gibco, Paisley, Scotland, UK) and fixed using 4% paraformaldehyde (Sigma-Aldrich MO, USA). Primary antibodies were applicated for overnight incubation. The expression of ICAM (BD Pharmingen, cat. 555511, San Jose, CA, USA), COX-2 (Thermo Scientific, cat. 35-8200, Waltham, MA, USA), NF-κB (Abcam, cat. ab16502, Cambridge, UK), Phospho-Histone γH2A.X (Ser139) (Cell Signalling, cat. 80312, Ma, USA). Then the proper secondary antibody with the fluorophore was added for 45 min: Alexa Fluor 488-conjugated Goat-anti-mouse (cat.115-545-003), AlexaFluor488-conjugated Goat-anti-rabbit (cat. 111-545-003) or Cy3-conjugated goat anti-rabbit (cat. 111-165-003, Jackson ImmunoResearch, West Grove, PA, USA). Nuclei were visualized using Hoechst 33342 (Invitrogen, Waltham, MA, USA). Images were acquired using a monochromatic AxioCam and AxioObserver 22 D1 inverted fluorescent microscope (Carl Zeiss Jena, Oberkochen, Germany). Each of the biological experiments included 3 well technical replicates, and a minimum of 5 images were collected from each well, or CQ1 Confocal Quantitative Image Cytometer (Yokogawa, Tokyo, Japan). The ratio of immunopositive cells to the total number of cells for COX-2, ICAM, and NFκB proteins was analyzed automatically using Columbus v. 2.4.2 software (Perkin Elmer, Waltham, MA, USA).
