# Supplementary figures and images for: Phylloquinone improves endothelial function, inhibits cellular senescence, and vascular inflammation
Source: GeroScience. 2024 Jul 9;46(5):4909–35. doi: 10.1007/s11357-024-01225-w (PMC11336140; doi:10.1007/s11357-024-01225-w)

scrambled control

Merge

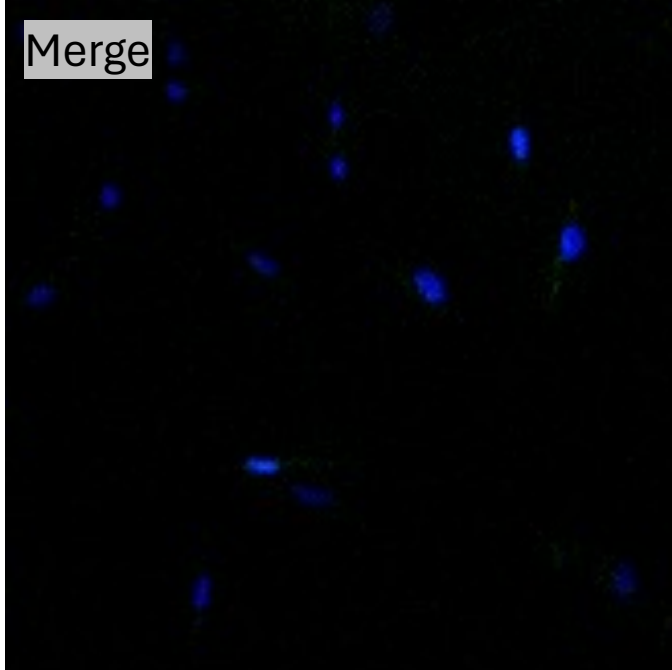

$\gamma$ H2A.x

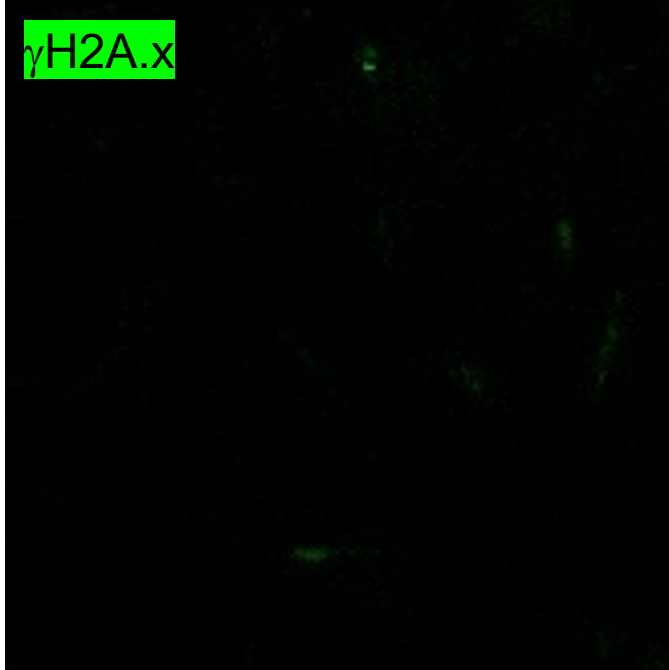

DAPI

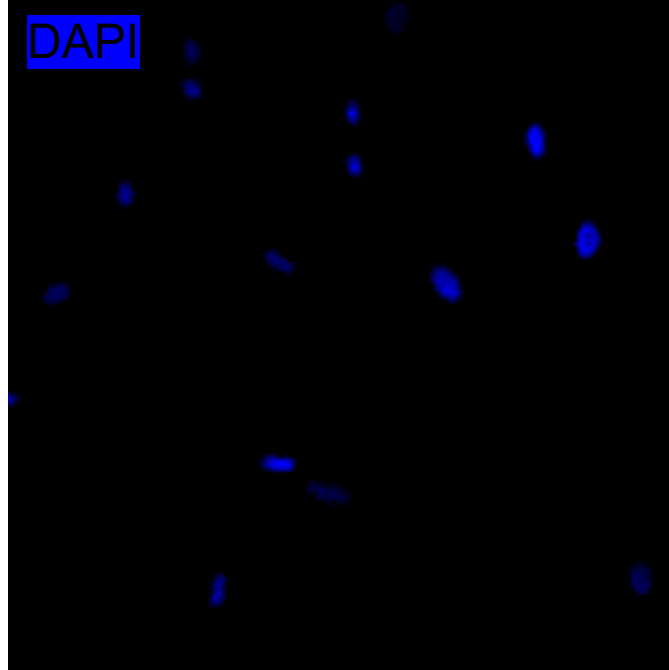

FACE-1 KO

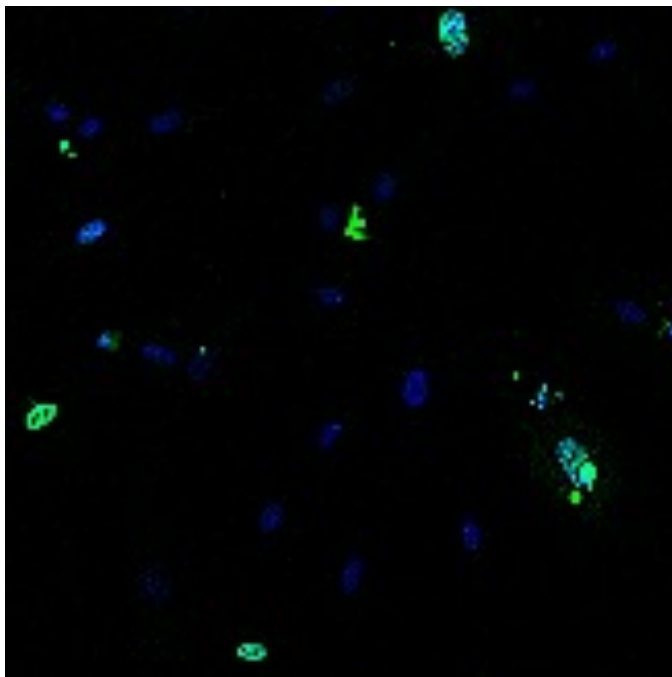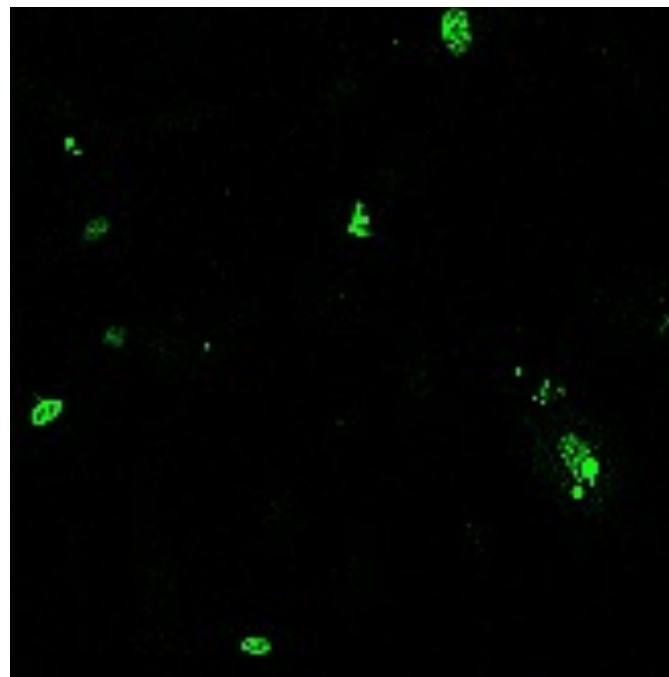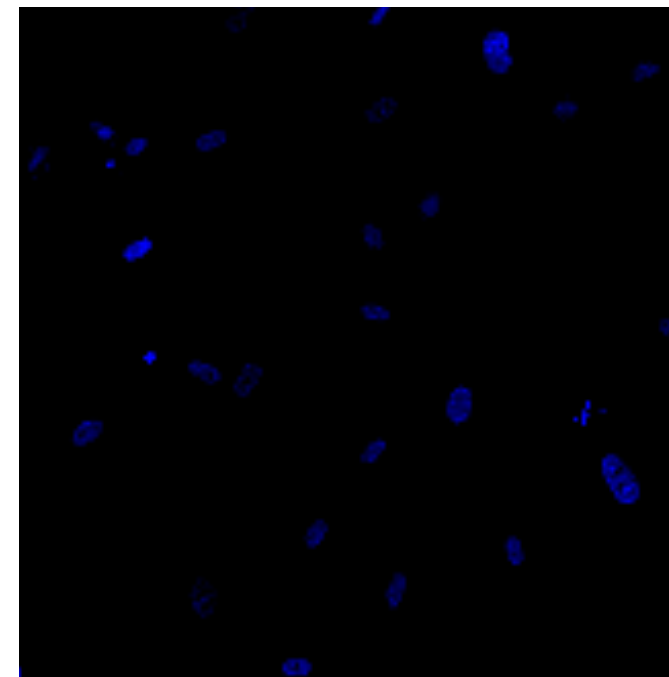

Supplement: Supplementary file 2 — Supplementary Figure 1 The effect transfection of the 04/35F/11A vascular smooth muscle cells with the scrambled control siRNA vs FACE-1 siRNA on phosphorylated yH2A.x expression (PDF 241 KB) [file 11357_2024_1225_MOESM2_ESM.pdf]
